# CRISPR/Cas9-mediated generation and analysis of N terminus polymorphic models of β_2_AR in isogenic hPSC-derived cardiomyocytes

**DOI:** 10.1016/j.omtm.2020.10.019

**Published:** 2020-10-27

**Authors:** Alexander Kondrashov, Nurul A.N. Mohd Yusof, Alveera Hasan, Joëlle Goulding, Thusharika Kodagoda, Duc M. Hoang, Nguyen T.N. Vo, Tony Melarangi, Nazanin Dolatshad, Julia Gorelik, Stephen J. Hill, Sian E. Harding, Chris Denning

**Affiliations:** 1 Division of Cancer and Stem Cells, University of Nottingham Biodiscovery Institute, University Park, Nottingham NG7 2RD, UK; 2 National Heart and Lung Institute, Imperial College, London W12 0NN, UK; 3 Centre of Membrane Proteins and Receptors (COMPARE), Universities of Birmingham and Nottingham, Midlands, UK; 4 Division of Physiology, Pharmacology and Neuroscience, School of Life Sciences, University of Nottingham, Nottingham, UK

## Abstract

During normal- and patho-physiological situations, the behavior of the beta2-adrenoreceptor (β_2_AR) is influenced by polymorphic variants. The functional impact of such polymorphisms has been suggested from data derived from genetic association studies, *in vitro* experiments with primary cells, and transgenic overexpression models. However, heterogeneous genetic background and non-physiological transgene expression levels confound interpretation, leading to conflicting mechanistic conclusions. To overcome these limitations, we used CRISPR/Cas9 gene editing technology in human pluripotent stem cells (hPSCs) to create a unique suite of four isogenic homozygous variants at amino acid positions 16(G/R) and 27(G/Q), which reside in the N terminus of the β_2_AR. By producing cardiomyocytes from these hPSC lines, we determined that at a functional level β_2_AR signaling dominated over β_1_AR . Examining changes in beat rates and responses to isoprenaline, Gi coupling, cyclic AMP (cAMP) production, downregulation, and desensitization indicated that responses were often heightened for the GE variant, implying differential dominance of both polymorphic location and amino acid substitution. This finding was corroborated, since GE showed hypersensitivity to doxorubicin-induced cardiotoxicity relative to GQ and RQ variants. Thus, understanding the effect of β_2_AR polymorphisms on cardiac response to anticancer therapy may provide a route for personalized medicine and facilitate immediate clinical impact.

## Graphical Abstract

**Figure:**
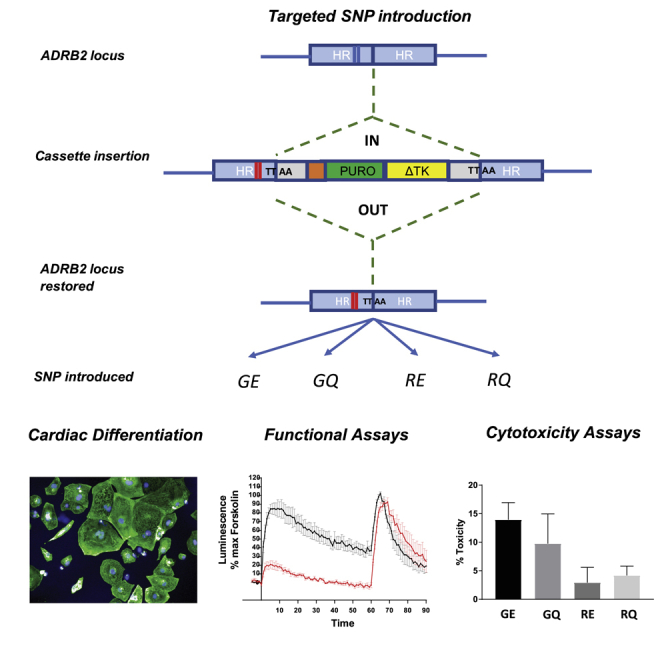

## Teaser

During normal- and patho-physiological situations, the behavior of beta2-adrenoreceptor (β_2_AR) is determined by its polymorphic variants. A human-based isogenic model system represents a promising tool for systematic analysis of mechanisms of β_2_AR variant-mediated cellular response under normal and stressed conditions. This allows important subtleties of polymorphisms in β_2_AR to be unraveled.

## Introduction

The beta-adrenergic receptors (βARs) are members of the G-protein-coupled receptor (GPCR) superfamily. They have important roles in various diseases, including cardiovascular disease and asthma, hence are common protein targets for therapeutic intervention.^1^^,^^2^ While there are 3 βARs (β_1_AR, β_2_AR, and β_3_AR), β_3_AR is not cyclic AMP (cAMP)-dependent, and its role in acute contractile changes is unclear. In cardiomyocytes, both β_1_ and β_2_ receptors are often found together but are spatially separated and co-localized with different effector complexes.^3^^,^^4^ Thus, both β_1_AR and β_2_AR receptors can be coupled to adenylate cyclase through the Gs subunit of trimeric G-protein. However, only β_2_AR can activate an alternative Gi-mediated pathway.^5^^,^^6^ This alternative coupling means β_2_AR can counteract proapoptotic signaling mediated by β_1_AR^7^ after prolonged catecholamine stimulation, and hence the β_2_AR variant is often considered as cardioprotective.^8^

Multiple single-nucleotide polymorphisms exist in *ADRB2*, the gene that encodes β_2_AR.^9^ Two of the most common variants cause nonsynonymous substitutions at positions of 16 (Gly to Arg; G to R) and 27 (Glu to Gln; E to Q) in the N terminus of β_2_AR.

Numerous genetic association studies have suggested that these variants may modulate the risk of developing heart failure, asthma and airway hyper-responsiveness, protection from traumatic or septic shock, obesity, and cancer, where β_2_AR plays a central role.^10^, ^11^, ^12^, ^13^, ^14^ Nevertheless, data often lead to conflicting mechanistic explanations, likely relating to the complex and heterogenic nature of the *ADRB2* locus.^15^^,^^16^

The impact of the polymorphisms in modulating functional responses of β_2_AR to stimulation with agonists has also been investigated. To this end, the approaches employed include using primary cell lines derived from genotyped individuals, such as lymphoblasts or airway smooth muscle cells,^17^^,^^18^ and stable or transient receptor overexpression in HEK293 or Chinese hamster ovary (CHO) immortalized lines.^19^^,^^20^ Although the polymorphic variants caused changes such as agonist-promoted receptor desensitization and downregulation,^19^ and trafficking in cytoplasm and phosphorylation pattern of the β_2_AR,^20^^,^^21^ the results were not always in concordance with each other.^19^^,^^20^ These model systems are limited by the high level of transgene expression and/or variability in the background genotype, factors known to cause phenotypic variability that exceeds the impact of the polymorphic modification.^22^^,^^23^ This has led to the notion that the gold standard is to engineer isogenic sets of cell lines, wherein only the polymorphism of interest is changed within an otherwise constant genetic background.

In the current study, we present and functionally characterize a unique set of homozygous isogenic human pluripotent stem cell (hPSC) lines and derived cardiomyocytes carrying four β_2_AR N-terminal polymorphic combinations at positions 16 and 27, namely Gly16-Glu27 (GE), Gly16-Gln27 (GQ), Arg16-Gln27 (RQ), and Arg16-Glu27 (RE).

## Results

### Engineering of isogenic hPSC lines carrying GE, GQ, RQ, and RE variants in the β_2_AR

**Figure 1:**
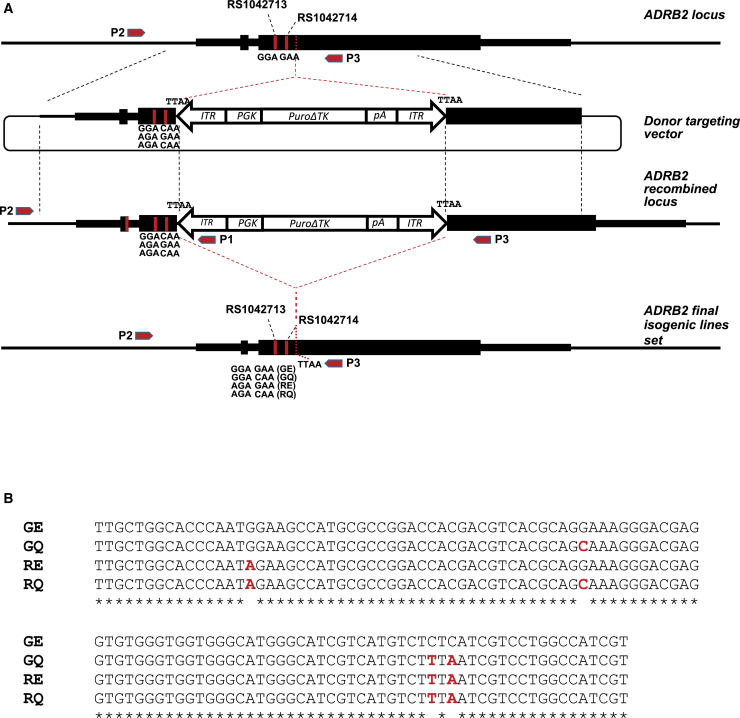
Generation of CRISPR-Cas9/PiggyBac-mediated isogenic models for *ADRB2* polymorphisms (A) Targeting strategy, demonstrating (1) relative polymorphic positions rs1042713 and rs1042714 are represented by homozygous GGA and GAA within HUES7 *ADRB2* gene locus. The thin line corresponds to genomic area surrounding *ADRB2* gene, and black boxes indicate main ORF as well as uORF situated within 5′UTR. 5′ and 3′UTRs are also shown (medium size black box); position of targeting cassette integration/excision point is indicated by dashed red line. (2) Donor targeting vector: thin circular line corresponds to background targeting plasmid, *PuroΔTK* selection cassette flanked by 5′/3′ *PiggyBac* terminal repeat (TR) regions (*ITR*, arrow type boxes) as indicated. *PGK* promoter and bGH polyadenylation site (pA) are also highlighted; regions of 1 kb of each left and right *ADRB2* flanks are bordered by dashed lines. (3) *ADRB2* recombined locus: intermediate step after integration of selection cassette into desired position by homologous recombination. (4) *ADRB2* final isogenic lines: modified *ADRB2* locus, restored after excision of resistance cassette by *PiggyBac* transposase and carrying modified *ADRB2* gene with introduced polymorphic variants. (5) Red arrows indicate positions of primers used for genotyping. (B) Aligned sequences surrounding sites of genome-editing manipulations including positions 46 and 79 and positions 124 and 126 (note resulting TTAA sequence). Original sequencing results confirming correct editing are shown in Figure S1.

To study the influence of polymorphisms on cardiomyocyte function, we created 4 isogenic homozygous variants within HUES7, a human embryonic stem cell (hESC) line,^24^ at positions 16 and 27 of the β_2_AR protein (46 and 79 of the *ADRB2* coding sequence). This entailed coupling CRISPR/Cas9 and *PiggyBac* approaches in a two-step “in-out” seamless strategy, which we recently published^22^ (Figure 1A). Targeting vectors contained the polymorphic variants within the left arm of homology, while a puroΔTK positive-negative selection cassette was flanked by *PiggyBac* transposon terminal repeats (Figure 1A; Figure S1A).

The targeting process in hPSCs followed sequential steps: transfection with vectors and selection using puromycin to isolate drug-resistant clones (the “in” step; Figure S1B), which were then transfected with transposase to excise the selection cassette via recombination of *PiggyBac* sequences, thus reconstituting the locus and rendering the cells insensitive to ganciclovir (the “out” step; Figure S1B). The use of *PiggyBac* transposon targeting vector requires the presence the TTAA integration/excision sequence. Since the nearest TTAA sequence to polymorphic positions exceeded the limit of efficient gene conversion (748 bases away from gRNA-A1 cleavage site; Figure S1A), we used codon redundancy to make a synonymous change in Leu42-Ile43 (CTCATC to TTAATC) (Figure 1A; Figure S1A).

Clones for the four β_2_AR variants (GE, GQ, RQ, and RE) were verified by PCR genotyping, and “scar-free” fidelity of the locus after *PiggyBac* cassette excision was confirmed by sequencing (Figures S1C and S1E). It was important to discount the possibility of multiplications of targeted sequences and of large deletions in the second allele, which has been reported by others^25^ and can cause allelic dropout in PCR screening approaches. qPCR analysis showed similar values between the variants (Figure S2E). In addition, we exploited the suboptimal excision properties of transposase to demonstrate that both alleles could be detected by PCR in clones where excision of *PiggyBac* had only occurred in one allele, hence giving a size differential (Figure S3).

We also used primers designed to detect vector elements to ensure random sequences were not integrated elsewhere in the genome (Figures S1F and S2E). qPCR with targeting vector-specific primers did not detect the presence of randomly integrated plasmids into the genome of these isogenic cell lines (Figure S1D). This confirmed cassette excision and seamless restoration of the *ADRB2* locus, concurrent with incorporation of the different polymorphisms within the HUES7 genetic background, thereby providing a resource for characterization, cardiomyocyte differentiation, and phenotypic analysis.

### Characterization of isogenic β_2_AR variant lines

The isogenic lines displayed properties consistent with pluripotency relating to high nuclear to cytoplasmic ratio; compacted morphology; expression of OCT4, NANOG, and SOX2 markers; normal 46, XY chromosome complement; and constant proliferation through serials passages (Figures S2A–S2D). Directed differentiation as monolayers, via a protocol we published previously,^22^ showed all lines could produce cultures containing >90% hPSC cardiomyocytes, based on staining for alpha-actinin (Figure S4A).

Using this differentiation strategy, we also sought to evaluate expression levels of a selected gene set to establish the time point at which future studies were conducted. Over a 66-day time course of differentiation, quantitative real-time PCR showed expression level stabilized by day 30–40 for *ADRB1*, *ADRB2*, *GRK2*, *GRK5*, and *ARRB2* (Figure S4B). Interestingly, the profile of *ARRB1* appeared to differ between the β_2_AR variant lines, but these levels also stabilized after day 30. Thus, all further experiments were conducted using hPSC-cardiomyocytes of day 30–40 differentiation.

Signaling via the β_2_AR functions through adenylate cyclase to produce the second messenger, cAMP.^26^ Therefore, to assess whether cardiomyocytes from isogenic lines carrying polymorphic receptor isoforms possessed β_2_AR-specific pharmacology, we used a radioactive cAMP accumulation assay. In this assay, the addition of 3-isobutyl-1-methylxanthine (IBMX), a phosphodiesterase inhibitor, inhibits the typical and rapid intracellular breakdown of cAMP. To allow detection of weaker responses (e.g., β_1_AR) we extended incubation time to 5 h.

**Figure 2:**
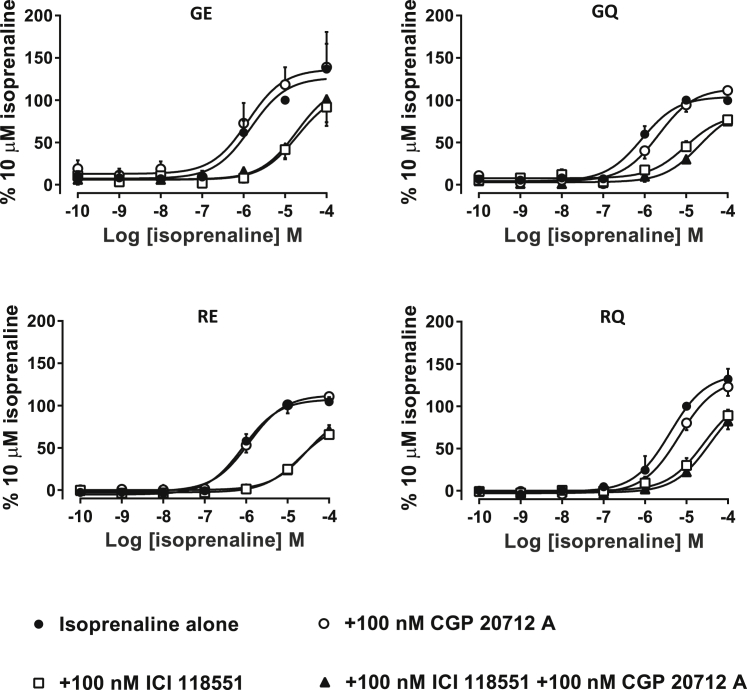
β_2_AR-specific pharmacology preserved in cardiomyocytes derived from isogenic β_2_AR polymorphism-specific cell lines ^3^H-cyclic AMP accumulation in response to isoprenaline and in the presence or absence of subtype-specific antagonists (β_1_AR-CGP 20172A, β_2_AR-ICI 118551). Cardiomyocytes show very specific β_2_AR pharmacology, with nearly absent β_1_AR response. Data points are presented as means ± SEM. Experiments were performed in triplicate (n = 5–7 measurements per condition, depending on treatment).

**Figure 3:**
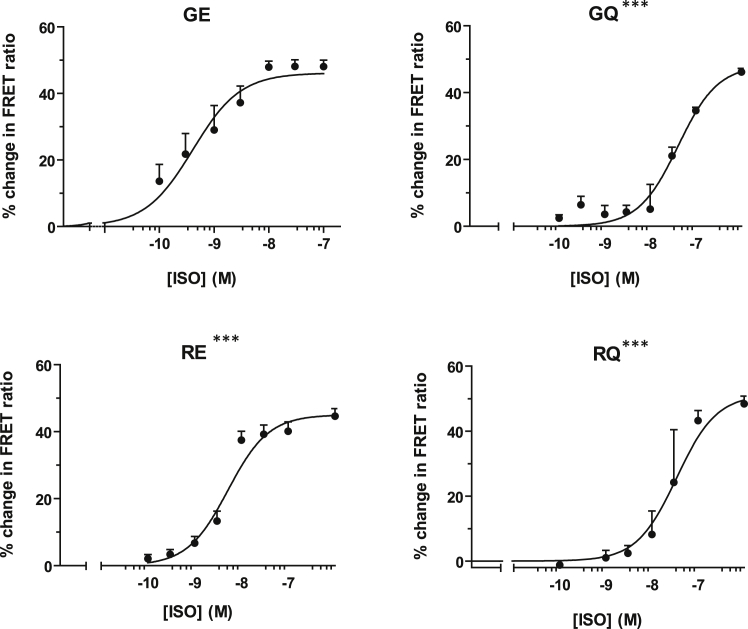
Concentration-response curves to increasing concentrations of isoproterenol (ISO) The EC_50_ of the 4 β_2_AR isoforms in hiPSC-CM were calculated in response to β_2_AR stimulation with isoprenaline in the presence of 100 nM CGP20172A and measured using real-time FRET microscopy. Upon obtaining a stable baseline after β_1_AR inhibition, β_2_ARs were stimulated by stepwise increments of isoprenaline concentration. The response was recorded until a plateau was reached for each increment, typically a maximum of 10 min, before the addition of the next isoprenaline solution. Overall time of experiment, including baseline recording, was 60 min. cAMP response normalized to NKH. FRET values are presented as means ± SEM. N values are as follows (n/N, where n = experiments and N = batch preparations): GE (4/2), RQ (3/2), RE (9/4), and GQ (4/4). Significantly different from GE, ∗∗∗p < 0.001, F-test.

In all four lines, isoprenaline, a non-selective β_2_AR competitive agonist, produced a concentration-dependent increase in 3H-cAMP accumulation (Figure 2). In the presence of 100 nM of ICI 118551, a selective β_2_AR antagonist, a rightward shift in the concentration response curve was observed. However, no shift was detected when CGP 20712A, a selective β_1_AR antagonist, was used. The low half-maximal effective concentration (EC_50_) observed here (as compared to data shown in Figure 3) is most likely due to the timescale of the experiment. During the 5-h cAMP measurements, the β_2_AR will have undergone significant desensitization, hence shifting the EC_50_ values to higher agonist concentrations and bringing them close to the agonist equilibrium dissociation constant (K_D_) values.^27^ These data indicated that β_2_AR-mediated pharmacological responses predominate over β_1_AR in cardiomyocytes from these isoform-specific hPSC lines. Although we detected expression of both *ADRB1* and *ADRB2* mRNAs, only β_2_AR presents detectable activity and therefore is the dominant receptor type. Our data on dominance of β_2_AR over β_1_AR activity in hPSC-cardiomyocytes is in concordance with previous findings.^28^

Collectively, we created an isogenic set of *ADRB2* gene edited variants that retain characteristics of hPSCs. This includes high-efficiency differentiation into cardiomyocytes, in which gene expression stabilized by day 30–40 to give a specific β_2_AR cAMP accumulation pharmacological profile.

### Sensitivity of hPSC-cardiomyocytes to isoprenaline is dependent on β_2_AR polymorphisms

To determine whether specific polymorphic combinations influence immediate cAMP production in hPSC-cardiomyocytes, we used real-time fluorescence resonance energy transfer (FRET) microscopy to calculate the EC_50_ in response to β_2_AR stimulation.^29^^,^^30^ Cumulative increases in concentration of isoprenaline (0.1–100 nM) were used as an agonist, allowing β_2_AR responses to be isolated by preincubating with the β_1_AR blocker (CGP 20712A, 100 nM). GE showed the highest sensitivity to β_2_AR stimulation, with an EC_50_ of 1 nM, similar to RE, yet significantly different (4 nM; Figure 3). Substitution of E27 to Q27 (i.e., GE to GQ and RE to RQ) showed much more dramatic reduction in sensitivity compared to both GE and RE variants, with an EC_50_ of 31 nM (GQ) and 29 nM (RQ). These data suggest that β_2_AR polymorphism in position 27 (E to Q) has a strong effect on immediate cAMP response to ligand stimulation in hPSC-cardiomyocytes, whereas position 16 (G to R) plays a more minor role. However, stimulation with isoprenaline might differentially affect immediate changes in expression of variant-specific β_2_ARs, which in turn could impact the kinetics of cAMP production.

### Differential hPSC-cardiomyocyte contractility in β_2_AR variants in normal and stressed conditions

**Figure 4:**
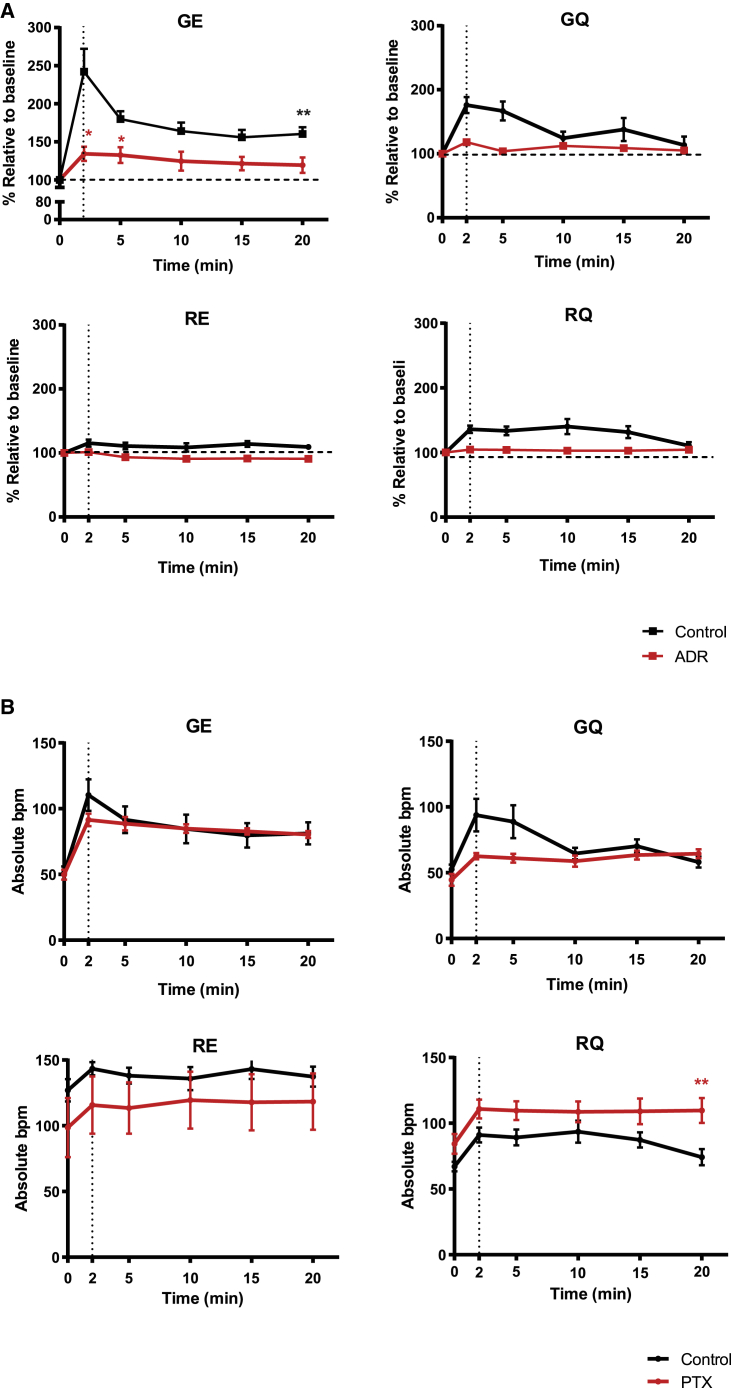
Contractility response to isoprenaline in unstimulated or stressed β_2_AR variant-specific cardiomyocytes (A) Increase in beating rate over basal with 0.01 μM isoprenaline in the presence of 300 nM CGP20172A. Data presented as mean ± SEM. N values (n/N, where n = experiments and N = batch preparations): GE (6/6), GQ (5/5), RQ (6/6), and RE (7/7). Repeated-measures ANOVA. Only GE above basal at 20 min, ∗∗p < 0.01; β_2_AR response after 20 min pre-treatment with high adrenaline (ADR, red line). (B) Increase in beating rate from baseline with isoprenaline in the presence of β_1_AR-blocker CGP20172A in control and PTX-treated cells for each isogenic line. Data shown as absolute beats per minute (bpm) and presented as mean ± SEM. N values (n/N, where n = experiments and N = batch preparations) for PTX GE (7/6), GQ (5/5), RQ (6/6), and RE (7/7). Significantly different from control, ∗∗p < 0.01.

We then decided to evaluate whether differences observed between hPSC-cardiomyocytes from β_2_AR variant-specific lines in immediate cAMP response associated with altered contractile behavior under normal and stressed conditions. Monitoring beating rate following β_2_AR stimulation with isoprenaline in the presence of the β_1_AR blocker CGP 20712A (300 nM) showed GE rate increased by 2.3-fold within the first 2 min. This rate reduced by 5 min and stabilized at 1.7-fold higher than that of basal contractility by 20 min post-treatment (Figure 4, Control). In comparison, GQ cardiomyocytes had a contractility rate of 1.8-fold greater than basal rate when treated with isoprenaline, which steadily decreased over the 20-min isoprenaline stimulation before returning to basal levels. The RQ cell line showed the least increase in contractility after isoprenaline treatment, a 1.4-fold increase from that of basal. Surprisingly, RE cardiomyocytes showed no significant change in contractility from baseline upon isoprenaline stimulation.

To assess the effect of β_2_AR stimulation on beating rate during cardiac stress, hPSC-cardiomyocytes were subjected to a simulated adrenaline shock model.^31^ Thus, all 4 lines were challenged by pre-treatment with 1 μM adrenaline to initiate βAR desensitization or Gi-switching, and then cells were exposed to β_2_AR stimulation with isoprenaline in the presence of β_1_AR blocker CGP 20712A. Cardiomyocytes expressing the β_2_AR GE variant increased beat rate to 1.2-fold basal at 2 min, which remained raised throughout the 20-min isoprenaline treatment. Contractility of GQ cells increased to 0.2-fold at 2 min but returned to basal levels after 20 min of isoprenaline treatment. Interestingly, adrenaline pre-treatment led to the change in cellular contractility of RQ as well as RE cells being completely suppressed (Figure 4A).

To reveal the differences in tonic effect of Gi-switching in depressing beating rate, we treated hPSC-cardiomyocytes with pertussis toxin (PTX), an agent that abolishes the negative chronotropic response of muscarinic or adenosine agonists.^32^ While no significant Gi effect was seen for GE, GQ, and RE, we found a significant increase in beating rate in the RQ variant in PTX-treated cells compared to control (t[10] = 3.583, p = 0.005) over the 20 min of β_2_AR stimulation with isoprenaline (Figure 4B).

These results showed that GE variant of β_2_AR mediated the strongest effect on cardiomyocyte contractility upon stimulation with isoprenaline. While all combinations of polymorphisms demonstrated differing degrees of desensitization under stress conditions, only the RQ combination responded positively to PTX treatment. Therefore, differential contractility responses observed in β_2_AR variant-specific lines could only partially be explained by differences in cAMP response (Figure 3) or coupling to Gi (Figure 4B)

### Downregulation of cAMP response is polymorphism dependent in hPSC-cardiomyocytes

**Figure 5:**
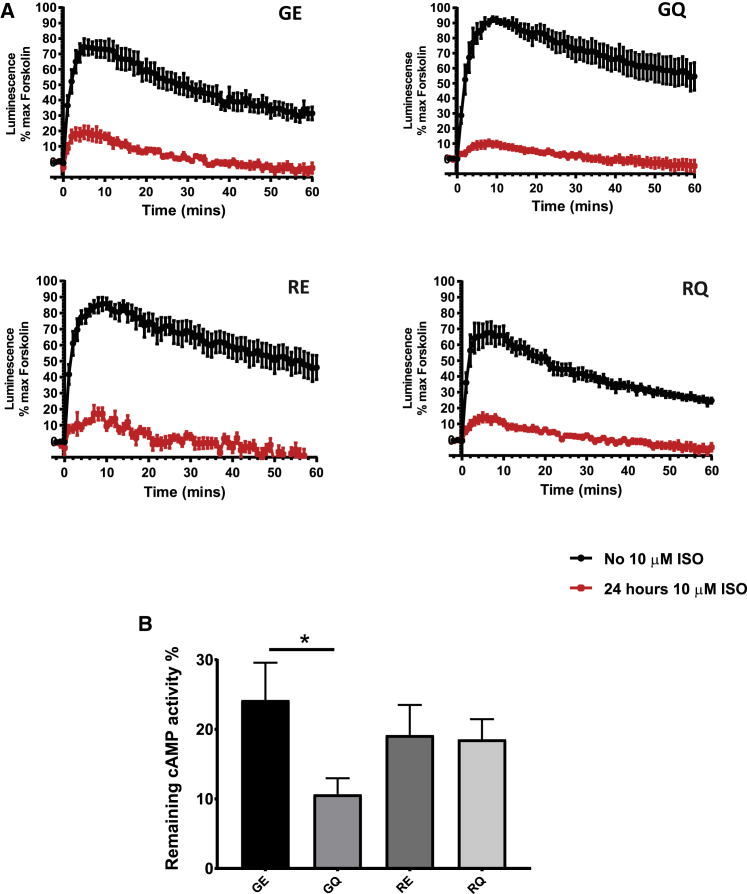
Downregulation of β_2_AR cAMP production in response to prolonged exposure to isoprenaline Cardiomyocytes from GE, GQ, RE, and RQ lines were transfected with pGloSensor 20F plasmid and after 48 h were incubated with 10 μM isoprenaline for 24 h. After washing, cells were re-challenge with 1 μM of isoprenaline and real-time cAMP response was measured. (A) Time course analysis during 1 h after re-challenge. (B) Maximum remaining cAMP response after 24 h exposure to 10 μM isoprenaline normalized to maximum untreated control (0 h). cAMP across multiple experiments. Data are presented as means ± SEM (n ≥ 7, two-tailed unpaired t test; ∗p = 0.0349).

Prolonged stimulation of β_2_AR with agonists leads to receptor downregulation.^33^ To understand whether polymorphic combinations affect the extent of receptor downregulation, we used real-time monitoring of cAMP production as a measure of β_2_AR activity. Cardiomyocytes from all variant-specific hPSC lines were transfected with Luc-RIIβB-Luc split-luciferase vector^34^ and exposed to a saturating concentration of isoprenaline (10 μM) for 24 h in order to promote receptor downregulation. Real-time measurement after wash and re-challenge with 1 μM isoprenaline showed significant downregulation of β_2_AR-mediated cAMP production in all lines when compared to control (Figure 5A; Figure S5A). There was a significant difference in percent cAMP induction for GE relative to GQ (Figure 5B). In addition, there was a trend, albeit non-significant, toward an increased level of downregulation in the GQ isoform relative to RE or RQ. This implies that Gly at position 16 coupled with Gln at position 27 shows the strongest downregulation of β_2_AR activity, compared to other polymorphic combinations (Figure 5).

### Desensitization of cAMP synthesis is dependent on polymorphic combinations

**Figure 6:**
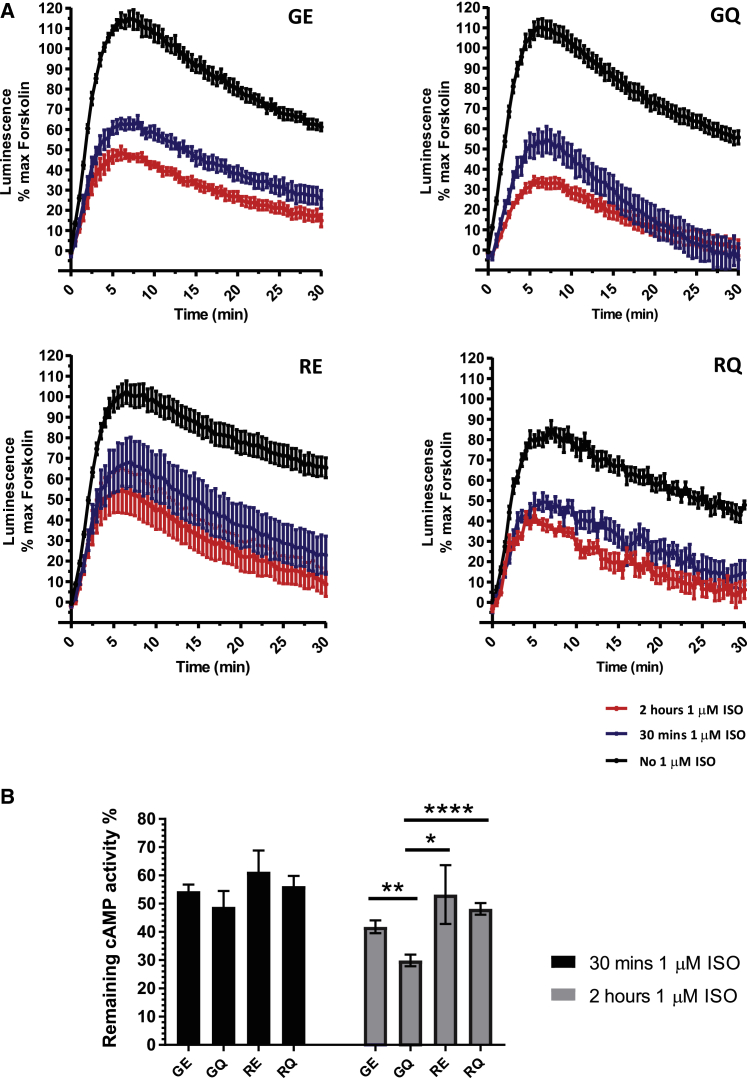
Isoprenaline-induced desensitization of β_2_AR cAMP production (A) Time course analysis of the effects of 30 min and 2 h of pre-treatment with 1 μM of isoprenaline versus sample treated with vehicle. (B) Comparison of remaining β_2_AR-mediated cAMP response to 1 μM isoprenaline after pre-treatment with isoprenaline for 0.5 or 2 h. Data are presented as means ± SEM (n ≥ 7, two-tailed unpaired t test, ∗p = 0.0133, ∗∗p = 0.0008, ∗∗∗∗p < 0.0001).

Previous reports using human lymphocytes homozygous for each haplotype group of β_2_AR have shown differential effect of polymorphisms on receptor desensitization.^35^ To determine whether this held true in the isogenic hPSC-cardiomyocyte model, we again relied on the same split-luciferase-based real-time system to measure cAMP response (see schematic in Figure S6). Cardiomyocytes were treated with 1 μM isoprenaline in the presence of CGP 20712A for a period of 30 min or 2 h to induce receptor desensitization. Subsequently, cells were washed, challenged a second time with isoprenaline, and then cAMP production monitored by measurement of luciferase activity. Percentage cAMP was calculated by normalizing to the peak induced by forskolin treatment, which causes nonspecific activation of adenylate cyclases and release of maximum potential cAMP (Figure 6; Figure S5).

Significant reduction of cAMP formation was seen at 30-min and 2-h time points for all β_2_AR polymorphic combinations compared to controls, but the magnitude of effect differed (Figure S5B). As before, calculation of the percentage cAMP induction showed a significant reduction in GQ relative to the other three haplotypes at the 2-h time point, consistent with the high sensitivity of this variant (Figure 6B; Figure S5B). Interestingly, GE also showed a tendency toward reduced percentage of cAMP induction relative to RE and RQ. Pre-treatment of hPSC-cardiomyocytes with CGP 20712A and ICI 118551 before addition of isoprenaline did not cause marked decreases in rate or cAMP production, though it should be noted that the concentrations of the blockers were well below those to induce inverse agonism.

These data suggest that β_2_AR variants carrying Gly16 show a faster desensitization dynamic compared to Arg16 (Figure 6B), and the observation that GQ has higher rate of desensitization is in agreement with work using human lymphocytes.^35^

### Differential toxicity of β_2_AR variant-specific cardiomyocytes to doxorubicin treatment

**Figure 7:**
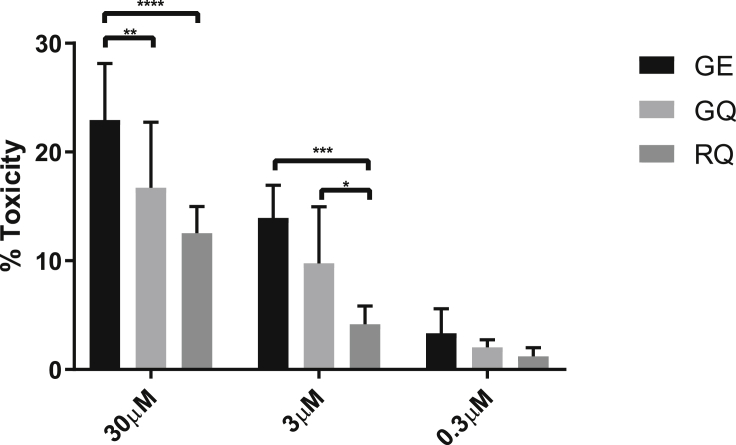
Doxorubicin-induced toxicity in GE, GQ, and RQ haplotype cardiomyocytes Cardiomyocytes from specific lines were treated with 30, 3, and 0.3 μM of doxorubicin for 24 h. Toxicity was evaluated by measurement of LDH release. Data are presented as means ± SEM (n ≥ 5, ∗p = 0.0199, ∗∗p = 0.0027, p∗∗∗ = 0.0003, ∗∗∗∗p = 0.0001 two-way ANOVA with Tukey’s multiple comparisons test).

The observation that different β_2_AR polymorphic isoforms are associated with altered functional responses prompted us to investigate whether this extended to variability in drug-induced cardiotoxicity.^36^^,^^37^ While it is known that β_2_AR is associated with sensitivity to doxorubicin,^38^^,^^39^ the influence of different polymorphic variants of the receptor has not been explored. Since the RE combination is almost absent in the human population, and therefore has no clinical significance, it was omitted from the following experiments. Using release of lactate dehydrogenase (LDH) as a measure of cell death,^37^^,^^40^ we found that all isogenic hPSC-cardiomyocyte lines showed substantial toxicity after 24 h of exposure to doxorubicin at 3 and 30 μM (Figure 7). However, the level of toxicity differed significantly, with GE and RQ showing highest and lowest sensitivity, respectively, and GQ falling between these two extremes. This observation is broadly consistent with other assays we have performed, particularly relating to the GE and RQ cardiomyocytes showing opposing responses, and implies β_2_AR isoforms warrant further investigation as predictive genetic markers of toxic response to drugs such as doxorubicin.

## Discussion

In an attempt to control the limitations of non-physiological expression and variable background genotype, we used CRISPR/Cas9 gene editing approaches to create an isogenic suite of β_2_AR polymorphisms in the N terminus of the protein within hPSCs. This afforded the opportunity to produce a renewable resource of functional hPSC-cardiomyocytes for direct inter-comparison, hence determining the impact of the GE, GQ, RQ, and RE homozygous polymorphisms at positions 16 and 27 of the β_2_AR. This demonstrated differential responses between the β_2_AR variants, particularly regarding sensitivity to isoprenaline, downregulation, desensitization, and doxorubicin-induced cardiotoxicity (DIC). This suggests that further understanding of the β_2_AR variants individuals express could be used to guide patient-specific precision medicine approaches.

This study has highlighted the advantages and disadvantages of hPSC-cardiomyocytes as a model system.^23^ A key benefit of this human-based system is its high genetic tractability, which provides opportunities to create isogenic suites of genetically normal cells that can be differentiated as a renewable resource to the target cell type of interest, while also retaining physiological levels of gene expression. In our case, we used a “scar-free” CRISPR/Cas9 and *PiggyBac* gene editing approach^41^ to modify *ADRB2*, which is a single exon gene with complex 5′ and 3′ regulatory regions.^42^ Nevertheless, we still needed to make subtle adaptations to enable the approach to be employed successfully. Thus, a synonymous change of a leucine codon CTC to TTA was needed to accommodate the requirements of the *PiggyBac* system.^41^ We did this to overcome the lack of endogenous TTAA at suitable proximity to the Cas9 cleavage site. In adopting this approach, we considered the potential impact on expression of *ADRB2* but reasoned the main two parameters, codon usage^43^ and location in the gene,^44^ were relatively risk free. The synonymous change to TTA produces a codon that is already naturally present in *ADRB2*, and it brings its usage frequency close to average observed for human (4.8 versus 7.2^45^^,^^46^). Given the fact that *ADRB2* is a low-expression gene, it is unlikely this introduction will place unnecessary demand on the translational machinery^47^ and affect expression level of β_2_AR. The TTA codon change we made was close to the start of the gene and considered unlikely to modify expression of *ADRB2* due to the effect on stop codon readthrough, since this effect is more commonly associated with changes in the 3′ region of the sequence controlling translational termination.^44^

The editing approach also allowed us to examine receptor density between different variants, while not unwittingly changing other parameters. For example, polymorphic variants in the 5′ UTR upstream open reading frame (uORF) of the *ADRB2* gene (minus 47 T>C, affecting amino acid at position 19 in the β_2_ upstream peptide [BUP]), has been found to be in strong linkage disequilibrium with positions 16 and 27 and influence expression of the β_2_AR protein.^16^^,^^42^ Because all the lines we created have the same variant at position minus 47, we can rule this out as a confounding factor in our interpretation of the results.

An unexpected challenge was the low expression levels of β_2_AR in hPSC-cardiomyocytes. Our initial attempts to measure receptor density by using radioligand binding assay failed, probably due to low level of expressed protein. The signal/noise ratio was not sufficient to provide scope to extract reliable information on specific binding in the presence and absence of our beta-adrenergic antagonists (Figure S7). Our own work (unpublished) and that of others demonstrates that existing β_2_AR antibodies lack both sensitivity and specificity to be useful in protein detection in cells with native low levels of β_2_AR expression.^48^^,^^49^ Thus, where immunostaining is performed, overexpression approaches are used to raise signal above background levels. We therefore used differences in maximal cAMP response to isoprenaline as an indirect measure of receptor abundance (Figure S8). The lower density of Arg16-containing variants has been reported before;^50^, ^51^, ^52^ in our case, RQ also showed reduced cAMP max response compared to GE or GQ variants, which can be explained by lower receptor density of RQ.

The potential influence of 5′UTR polymorphisms on receptor density^42^ in our model can be excluded since all variants were expressed under the same 5′UTR. An alternative explanation^53^ could be that some fraction of the β_2_AR population is already desensitized under normal conditions due to spontaneous/constitutive activity, a known characteristic of β_2_AR.^54^^,^^55^ It is possible that different polymorphic variants possess differing degrees of spontaneous activity. An important question for the future will be to include β_2_AR in heterozygous or heterozygous/homozygous configurations, since most possible combinations exist in the human population. This was beyond the scope of our study, because conducting a study with the numerous variations and power required, while also accounting for potential subtleties of different allelic-specific expression patterns, will make this a considerable undertaking. Indeed, this notion is supported by our observation of subtle differences in responses between homozygous lines, suggesting that only high powering of analysis across several heterozygous lines in parallel would be sufficient to unveil meaningful differences.

Analyzing response to isoprenaline by measuring the level of cAMP production showed several polymorphism-related differences. We observed reproducible stronger downregulation of agonist-induced cAMP production in GQ-carrying cardiomyocytes after both prolonged and short exposure to isoprenaline. There was a trend to stronger desensitization after isoprenaline exposure of isoforms with Gly16, both 30-min and 2-h time points. At 2 h, the GQ variant was desensitized to a greater extent than all other haplotype variants. The same tendency remained after prolonged 24-h exposure to a supramaximal concentration of isoprenaline when the GQ variant again showed the lowest cAMP response to β_2_AR stimulation, which indicated its high downregulation potential. Similar results, in terms of a greater degree of desensitization to GQ-induced cAMP production at 2 h, were shown in the study by Oostendorp et al.^35^ The authors, however, could not observe any haplotype-related differences in desensitization at 30 min or 24 h of treatment.

Our hypothesis that β_2_AR variants would have an influence on cardiomyocyte survival following doxorubicin exposure is based on previously published data. First, *ADRB2* gene knockout mice become sensitive to exposure to doxorubicin,^38^ an effect that can be reproduced *in vitro* using fibroblasts isolated from these animals.^39^ Second, human induced pluripotent stem cell (iPSC)-derived cardiomyocytes were used to recapitulate the phenotype from patients with DIC features.^36^ RNA sequencing (RNA-seq) data from this work show unequal distribution of *ARDB2* polymorphic isoforms between affected patients and those not affected by DIC, with GE being absent from patients resistant to treatment. Interestingly, the GE variant showed the highest sensitivity to doxorubicin in our LDH-based assay. The strongest cardioprotective effect against doxorubicin treatment was seen on the RQ isoform.

Differential response to doxorubicin in our experiments could be explained by differential coupling of β_2_AR variants to Gi. Indeed, our contractility experiments indicate that different β_2_AR N-terminal polymorphism variants might affect Gi coupling of the receptor with RQ, which showed a stronger response to PTX treatment than the other polymorphic combinations (Figure 4B). In agreement with this notion, low association of the Gly16 variant of β_2_AR with Gi was recently evidenced by Huang et al.^50^ The authors used rat cardiomyocytes transduced with adenoviral vectors expressing polymorphic variants of β_2_AR receptor, as well as lymphocytes isolated from genotyped patient carriers. They concluded that the Gly16 variant of the receptor is Gi defective. The latter, however, is arguable, since most studies on coupling of β_2_AR with Gi were done on animal models, and positions 16 and 27 are evolutionarily conserved and occupied by Gly and Glu, respectively.

Notwithstanding these benefits, the hPSC-cardiomyocyte system does have limitations. The differentiated cardiomyocytes produced reach only a limited level of maturity, and this has been well documented in the literature.^23^^,^^56^ In this study, we found that although levels of *ADRB1* and *ADRB2* mRNA were expressed in hPSC-cardiomyocytes and levels stabilized by day 30–40 of differentiation, β_2_AR was predominant at the pharmacological level. Others have described similar findings in hPSC-cardiomyocytes,^28^ and these differ from what is known for human adult cardiomyocytes.^57^ Moreover, the distribution of β_1_AR and β_2_AR differs with disease state, with β_2_AR typically residing in the t-tubules,^3^ structures which form poorly in hPSC-cardiomyocytes. Finally, creating polymorphic variants in hPSCs is recognized as one of the more challenging modifications to achieve, particularly when targeting 2 codons simultaneously to create four homozygous lines. This, coupled with clonal expansion, characterization, long differentiation timelines (30–60 days), costs, and complex phenotyping assays, means carrying this study out in more than one hPSC line was not possible. Indeed, to our knowledge at the time of writing, this is the first time such a set of lines has been created.

It is also acknowledged that, while the work we describe in this report uses cardiomyocytes of human genetic origin, all aspects are carried out *in vitro*. This provides the opportunity to carry out complex genetic modifications, and evaluate receptor function and signaling, and resulting phenotypes such as altered sensitivity to anticancer drugs. Nevertheless, the low receptor density in primary cells meant surrogate assays were required (e.g., for cAMP). Our unpublished data show that forced overexpression of *ADRB2* is not necessarily beneficial, because it compromises differentiation, survival into cardiomyocytes, and native ratios of the βAR isoforms (data not shown), although the overexpressed SNAP-tagged SNP variants showed receptor β_2_AR localization consistent with previous reports.^20^ Future work will be to repeat these experiments using endogenous expression levels. However, this will require careful Cas9/CRISPR engineering of SNAP-tagged SNP variants of β_2_ARs to ensure the complex elements in the 5′ and 3′ UTRs are not disrupted, since these are essential in regulating expression, processing, and membrane targeting.^16^^,^^42^

A complementary approach to the hPSC-cardiomyocyte model would be to make the modifications in mice, particularly given that positions 16 and 27 of *Adrb2* are occupied by Gly and Glu, respectively, hence similar to humans. This would allow the impact of the polymorphisms to be investigated *in vivo* using Cas9/CRISPR editing. However, species differences between mice and humans influence cardiac function and physiology,^56^ which extend to divergence β_1_:β_2_AR ratio, cAMP production, and Gi/Gs signaling.^58^

A strength of the hPSC-cardiomyocyte system is the potential to investigate polymorphisms that vary in their impact on phenotype and function from overtly disease-causing through to more subtle modifiers, and we have published data at both ends of the spectrum.^59^^,^^60^ While polymorphisms that modify phenotype are often common variations rather than mutations or rare polymorphisms and hence are often undetected in minor allele frequency (MAF)-related searches,^61^ they are important. Examples are D/I variant in ACE, associated with the COVID-19 pandemic,^62^ or Q41L in GRK5^63^ and G16R/E27Q in β_2_AR, associated with altered signaling in the heart.^50^ Given that β-blockers are one of the most prescribed medicines worldwide^64^^,^^65^ and the body of literature indicates the importance of the position 16/27 polymorphisms in β_2_AR,^1^^,^^2^^,^^9^, ^10^, ^11^, ^12^, ^13^, ^14^, ^15^^,^^50^^,^^65^, ^66^, ^67^ we elected to study these variants in the context of hPSC-cardiomyocytes.

In conclusion, we developed a human-based model system to allow systematic analysis of mechanisms of regulation of β_2_AR-mediated cellular response under conditions of toxic stress. This will allow important subtleties of polymorphisms in β_2_AR to be unraveled, including the life cycle of these receptors during stimulation, phosphorylation, and induction of alternative signaling pathways. The value of this is evident, given the double-edged sword nature of the β_2_AR. While in the heart the β_2_AR offers protection to cardiomyocytes by activating the Gi pathway,^31^ in the cancer setting it enhances invasiveness upon stimulation.^68^^,^^69^ Understanding the effect of β_2_AR polymorphisms on cardiac response to anticancer therapy would have immediate clinical impact and will help to choose more accurate therapy prescriptions. Having additional tools to evaluate the balance between these outcomes will be vital to define risk and benefit of drugs such as beta-blockers in precision medicine.

## Materials and methods

### Cell culture and reagents

All culture was at 37°C at 5% CO_2_ in a humidified atmosphere. Unless otherwise stated, all reagents were from Thermo Fisher. HUES7 hESCs were gifted by Chad Cowan and Doug Melton at the Harvard Stem Cell Institute. Culture was in E8 medium on Matrigel, although initial culture processes were done in hESC medium conditioned using mouse embryonic fibroblasts. Cell harvesting was done using 0.5 mM EDTA for passaging or with Accutase for freezing or before transfections.

### Construction of targeting and guide RNA (gRNA) expression vectors

*ADRB2* targeting vectors was constructed via Gibson assembly by using Gibson Assembly master mix (E2611S, NEB) as described previously.^22^ All primers used to create targeting vectors and gRNA-B5 plasmid are listed in Table S1. The general primers set to amplify left (primers P4 and P5) and right *ADRB2* homology arms (primers P6 and P7) from HUES7 gDNA and *PiggyBac* dual drug selection cassette (Puro-ΔTK, primers P8 and P9) from pMCS-AAT-PB:PGKpuro-ΔTK (obtained from Welcome Trust Sanger Institute plasmid repository, Kosuke Yusa laboratory, http://www.sanger.ac.uk/science/tools/piggybac-transposase-resources) are as outlined in Table S1. The reaction mixture was used to transform Top10 competent cells, and correct colonies were identified by restriction digestion analysis and sequencing. The original targeting vector therefore contained GE variant (pADRB2-PB-TARG-GE, GenBank: MT917127; complete sequence is shown in Figure S9) like that of the parental cell line. pADRB2-PB-TARG-RQ targeting vector was created by using the PCR-splice mutagenesis method as described previously.^70^ First, we generated two PCR products by amplifying fragment from pSIN-SNAP-ADRB2 plasmid,^71^ which encodes the RQ variant of the β_2_AR with primer pairs P10 and P11 and a fragment from the pADRB2-PB-TARG-GE template with P12 and P13 primers. Next, two fragments were fused together by PCR-slice reaction, and the resulting hybrid (resulting from PCR by flanking primers P10 and P13) was digested with NcoI restriction enzyme cloned into NcoI-digested pADRB2-PB-TARG-GE backbone, giving pADRB2-PB-TARG-RQ. GQ and RE variants were initially constructed within pSIN-SNAP-ADRB2 background by PCR-splice mutagenesis using the pairs of overlapping primers: P14 and P15 for RE, and P16 and P17 for GQ. The resulting pSIN-SNAP-ADRB2(RE) and pSIN-SNAP-ADRB2(GQ) were used to create pADRB2-PB-TARG-RE and pADRB2-PB-TARG-GQ in a similar way as for pADRB2-PB-TARG-RQ, as described above. We created two SNP-specific gRNA-vectors (gRNA-B5 to target position 48 and gRNA-A1 to target position 79 on *ADRB2* locus respectively; see Figure S1A). gRNA-A1 was described previously.^22^ To generate B5, we followed the recommendation of Addgene for constructing gRNA_Cloning Vector (Addgene, #41824)-based plasmids with Afl II digestion, following Gibson assembly reaction.^72^^,^^73^ Primers P18 and P19 were used to prepare an insert for Gibson reaction.

### Targeting of ADRB2 locus

We described previously our targeting approach for using a combination of *PiggyBac* and CRISPR/Cas9 techniques.^22^ In brief, for targeting experiments, HUES7 cells were seeded on Matrigel-coated 6-well plates at density 3 × 10^5^ cells per well. Twenty-four hours later, cells were transfected by 3.3 μg three CRISPR plasmid components (targeting vector, gRNA vector, and Cas9 expressing plasmid) by using FuGene HD transfection reagent according to manufacturer recommendations (Promega). After selection on puromycin (0.25 μg/mL), clones were manually picked up and PCR genotyped before selecting them for the transposase excision step. For cassette excision, selected clones were expanded and seeded on 6-well plates before transfection by transposase expression vector pCMV-hyPBase (obtained from Welcome Trust Sanger Institute plasmid repository, Allan Bradley laboratory, http://www.sanger.ac.uk/science/tools/piggybac-transposase-resources) as described above. Cells were reseeded to 10 cm plates and maintained in culture for 2 to 3 additional days to allow cassette excision by transposase and then exposed to 2 μg/mL of ganciclovir (Invivogene) for negative selection. Second-step selection clones were manually dissected and genotyped using primers shown in Table S1. Some clones had excision only from one allele and showed presence of cassette in remaining alleles, so were not chosen for further experiments. Nevertheless, these “incomplete excisions” confirm correct biallelic insertion of cassette during the first targeting step and absence of potential large deletions in alternative alleles, which can be the result of Cas9-mediated cleavage.^25^ Selected clones were also checked for absence of off-targets (see Figures S10 and S11) before performing experiments. We tested all candidates with potential to disrupt gene coding areas for both gRNA-A1 and gRNA-B5. All primers used are indicated in Table S1, and gene target names are presented in Figures S10 and S11. PCR conditions for off-target detection were as described previously.^22^

### Monolayer cardiac differentiation

The monolayer cardiac differentiation process was conducted as previously described.^22^ Briefly, the cell lines were first seeded at 40,000 cells/cm^2^ in a Matrigel-coated T25 flask and cultured in E8 medium until reaching 80% confluence. Once the confluence was reached, the culture was pre-conditioned using StemPro34 medium containing 1 ng/mL BMP4 (bone morphogenetic protein 4) and Matrigel (1:100) 1 day before starting the differentiation process. On day 0, the confluent culture was treated with StemPro34 medium supplemented with 10 ng/mL of BMP4 and 8 ng/mL of Activin-A for 48 h. On day 2 post differentiation, the medium was switched into RPMI medium with added B27 (minus insulin) supplement and 10 μM of KY02111 and XAV939 (KYX). On day 4, the medium was changed into RPMI supplemented with B27 and 10 μM of KYX. After 48 h, the medium was changed into RPMI/RB27 (RB27), and the differentiated cells were maintained in it. The signs of beating started from day 6 to day 8 after initiation of the differentiation process.

### Real-time PCR

Total RNA from cells of days 0, 20, 30, 40, 50, and 60 post cardiac differentiation was extracted using the NucleoSpin RNA purification system (Macherey-Nagel) according to manufacturer recommendation. cDNA was produced from 250 ng of total RNA by SuperScript III Reverse Transcriptase. The qPCR reaction was conducted by TaqMan Fast Advanced Master Mix on the Applied Biosystems 7500 Fast Real-Time PCR instrument. Relative expression of genes was calculated and expressed as 2^−ΔΔCt^. Expression values were normalized against expression of *GAPDH* as housekeeping gene. TaqMan assay IDs for *GAPDH*, *ADRB1*, *ADRB2*, *GRK2* (ADRK1), and *GRK5* are Hs02786624_g1, Hs02330048_s1, Hs00240532_s1, Hs00176395_m1, and Hs00992173_m1, accordingly.

### [3H]Cyclic AMP accumulation

The accumulation of tritiated cyclic AMP was assessed as described previously^27^ with the following modifications. Cardiomyocytes were plated at a density of 100,000 cells per well in 48-well plates and maintained until aged between 30 and 40 days in growth medium (RPMI 1640 medium-B27 supplement, Thermo Fisher). Cells were prelabeled with [3H]adenine (2 μCi/mL) for 2 h at 37°C in 1 mL/well growth medium. Following [3H] adenine removal and washing, either ICI 118551 or CGP 20712A (1 pM–10 μM) or 10 μM isoprenaline was added in growth medium containing the phosphodiesterase inhibitor IBMX (1 mM) to wells and allowed to incubate for 5 h prior to termination of reaction by the addition of 50 μL concentrated HCL.

### cAMP measurements via FRET

System set-up: FRET microscopy system consisted of an ORCA-ER CCD (Hamamatsu Photonics) camera attached to an inverted Nikon TE2000 microscope with a 100 W halogen lamp illuminator and EX436/20 excitation filter. The fluorescent light emitted from the object was split into YFP and CFP channels by Quad view beam splitter equipped with a DM455 dichroic mirror and 535/40 and 480/30 emission filters. Recorded images were analyzed by quantifying the relative ratio of YFP to CFP using Micro-Manager1.4 software package. The FRET ratio responses are presented at normalized data to the maximal cAMP released by activation of AC by NKH-477, a forskolin analog (Sigma-Aldrich). JetPRIME (Ployplus) transfection kit was used to transfect hESC-CM with the FRET-biosensor mTurquoise-Epac1-Venus-Venus (pTEV)^74^ utilizing the EPAC1 binding domain following manufacturer’s protocol. In brief, 200 ng of plasmid was diluted in 50 μL of jetPRIME buffer and vortexed for 10 s. To this, 2 μL of jetPRIME reagent was added and vortexed for 15 s before incubating at room temperature for 10 min. The transfection mixture was added to the cells in culture dishes containing 1.5 mL culture medium (RPMI + B27) and incubated for 24–72 h before use. For all FRET experiments, cells were seeded in MatTek dishes with a maximum volume of 2.5 mL. All reagents were prepared using a FRET buffer (NaCl 144 mM, HEPES 10 mM, MgCl_2_ 1 mM, KCl 5 mM [pH 7.4]) at the following concentrations: CGP 20712A 100 nM, isoprenaline 100 nM, NKH 10 μM.

### IonOptix system for contraction measurements

System set-up: cell contractility was measured using an IonOptix system, which consisted of a Nikon TE-200 inverted microscope and a MyoCam CCD Digital Camera (IonOptix) attached to the computer. Contractility recordings were analyzed using the IonWizard package. The behavior of β_2_AR on cellular contractility upon stimulation with a number of pharmacological agents was assessed. All reagents were prepared in Krebs-Henseleit (KH) solution at the following concentrations: CGP 20712A 300 nM, isoprenaline 10 nM, NHK 10 μM, adrenaline 1 μM, PTX 1.5 μg/mL. Cells were perfused with the solutions at 37°C for at a rate of 2 mL/min. Initial KH was to obtain a stable baseline beating rate to which treatment responses have been normalized. Protocols established to assess the contractile response of β_2_AR stimulation are as follows: control β_2_AR response: 15 min KH, 10 min selective β_1_ blocker CGP 20712A, followed by 20 min isoprenaline + CGP 20712A. *In vitro* stress model by pre-treating cells with adrenaline: 15 min KH, 20 min adrenaline, then 10 min CGP 20712A, then 20 min isoprenaline + CGP 20712A. To assess the influence of G_i_ proteins on cellular contractility: cells were pre-treated with PTX (1.5 μg/mL for 3 h at 37°C), before 15 min KH perfusion, 10 min CGP 20712A, and then 20 min isoprenaline + CGP 20712A. To assess the role of G_i_ proteins on cellular contractility during induced cardiomyocyte stress: cells were pre-treated with PTX as described above, then perfused with KH, 20 min adrenaline, 10 min CGP 20712A, and then 20 min isoprenaline + CGP 20712A.

### Downregulation assay

The β_2_AR isoform-specific (GE, GQ, RE, and RQ) cardiomyocytes, at density 30,000 cells/well, were transiently transfected with pGlosensor 20F (Promega) 0.1 μg plasmid DNA per well of a 96-well plate using Lipofectamine 3000. At 24 h after transfection, cells were changed into normal RB27 medium and allowed to maintain for 48 h. 24 h prior to the assay, cells were treated with 10 μM isoprenaline to stimulate downregulation. Cells were incubated at 37°C for 2 h in RB27 medium supplemented with 25 mM HEPES, 10% fetal bovine serum (FBS), and 2% GloSensor cAMP Reagent stock solution with β_1_AR blocker 200 nM CGP 20712A and with/without isoprenaline 10 μM. Each condition was performed in triplicate. Washouts were performed by removing GloSensor cAMP reagent mix/incubation medium, washing twice with fresh wash medium (RB27) without isoprenaline in total duration of 10 min, and then adding replacement Glosensor cAMP mix medium with CGP 20712A but without isoprenaline. Control wells received the same number of washes in the same volume of wash medium without isoprenaline. Luminescence was read on EnVision Plate Reader (PerkinElmer) in a temperature-controlled chamber at 37°C. Basal/baseline signal was measured for 20 min. Isoprenaline was added to wells at 1 μM final concentration to re-challenge the system, and luminescence was read for 60 min. Later, 10 μM forskolin was added to wells to stimulate total cAMP, and luminescence was read for another 30 min. Signal was measured by normalizing to maximum forskolin and subtracting the baseline. Three separate experiments, each done in triplicate, were quantitated and analyzed using Microsoft Excel and GraphPad Prism.

### Real-time measurement of β_2_AR desensitization

The β_2_AR variant (GE, GQ, RE, and RQ) cardiomyocytes, at density 30,000 cells/well, were transiently transfected with pGlosensor 20F (Promega) 0.1 μg plasmid DNA per well of 96-well plates using Lipofectamine 3000. At 24 h after transfection, cells were changed into normal RB27 medium and allowed to grow for 48 h. Cells were incubated at 37°C for 2 h in RB27 medium supplemented with 25 mM HEPES, 10% FBS, and 2% GloSensor cAMP Reagent stock solution with β_1_AR blocker 200 nM CGP 20712A. At the start of pre-incubation, final concentration of 1 μM isoprenaline was added at 2-h and 30-min time points. Each condition was performed in at least duplicate. Washouts were performed by removing GloSensor cAMP reagent mix/incubation medium, washing twice with fresh wash medium (RB27) without isoprenaline in total duration of 10 min, and then adding replacement Glosensor cAMP mix medium with CGP 20712A but without isoprenaline. Control wells received the same number of washes in the same volume of wash medium without isoprenaline. Luminescence was read on EnVision Plate Reader in a temperature-controlled chamber at 37°C. Basal/baseline signal was measured for 10 min. Isoprenaline was added to wells at 1 μM final concentration to “re-challenge” the system, and luminescence was read for 30 min. Later, 10 μM forskolin was added to wells to stimulate total cAMP, and luminescence was read for another 15 min. Signal was measured by normalizing to maximum forskolin and subtracting the baseline. Three to five separate experiments, each done in at least duplicate, were quantitated and analyzed using Microsoft Excel and GraphPad Prism.

### Cytotoxicity assays

For doxorubicin toxicity assay, cardiomyocytes were seeded on 96-well plates at 40,000 cells/well. Cells were treated with 30, 3, and 0.3 μM of doxorubicin or vehicle control for 24 h. Cell toxicity was evaluated by using Pierce LDH Cytotoxicity Assay Kit according to manufacturer recommendation.

### Statistical analysis

All statistical analysis was done in GraphPad Prism, version 5.0 or 7.0 (GraphPad Software) as follows: 3H-cyclic AMP accumulations were done as single experiments (n = 5–7) and performed in triplicate and mean logEC_50_ values compared using 1-way ANOVA with Tukey’s multiple comparison test; 2-way ANOVA with Turkeys multiple comparison test was used for LDH release experiment, and two-tailed unpaired t test was used for downregulation and desensitization assays. Statistical significance between the four haplotypes from FRET experiments were tested using the F-statistic; statistical significance between the four haplotypes from IonOptix contractility experiments were tested using repeated-measure ANOVA.

## Supplemental Information

Document S1. Figures S1–S11 and Table S1

Document S2. Article plus Supplemental Information
