# Supplementary material for: CRISPR/Cas9-mediated generation and analysis of N terminus polymorphic models of β2AR in isogenic hPSC-derived cardiomyocytes
Source: Mol Ther Methods Clin Dev. 2020 Oct 27;20:39–53. doi: 10.1016/j.omtm.2020.10.019 (PMC7733025; doi:10.1016/j.omtm.2020.10.019)
Supplement: Document S1. Figures S1–S11 and Table S1 [file mmc1.pdf]

OMTM, Volume *20*

Supplemental Information

CRISPR/Cas9-mediated generation and analysis of N terminus polymorphic models of β_2AR in isogenic hPSC-derived cardiomyocytes

Alexander Kondrashov, Nurul A.N. Mohd Yusof, Alveera Hasan, Joëlle Goulding, Thusharika Kodagoda, Duc M. Hoang, Nguyen T.N. Vo, Tony Melarangi, Nazanin Dolatshad, Julia Gorelik, Stephen J. Hill, Sian E. Harding, and Chris Denning

Table S1. Primers used in the current study

Primer	Use	Sequence
P1	genotyping	R 5' – CTAAATGCACAGCGACGGATTCGCGC -3'
P2		F 5' – GCTCGGGTGAGGCAAGTTCGG -3'
P3		R 5' – ATGGCAAAGTAGCGATCCAC -3'
P4	Left Flank	F 5' - GGTCGACGGTATCGATAAGCTTGATT TCGGAGTACCCAGATGGAGAC -3'
P5		R 5' - ACGCAGACTATCTTTCTAGGGTTAA AGACATGACGATGCCCATGC -3'
P6	Right Flank	F 5' - CAATATGATTATCTTTCTAGGGTTAA TCGTCCTGGCCATCGTGTTTGG -3'
P7		F 5' - ATCCCCCGGGCTGCAGGAATTCGATA GTCTTCCGTGCCTGGGAGGTC -3'
P8	PiggyBac Puro-ΔTK cassette	F 5' - CATCGTCATGTCTTTAACCCTAGAAA GATAGTCTGCG -3'
P9		R 5' - ACACGATGGCCAGGACGATTAACCCT AGAAAGATAATCATATTGTGACG -3'
P10	Construction of RQ, RE and GQ targeting vectors	F 5' – ACTGCGCGCCATGGGGCAACCCGGGAACGGCAG -3'
P11		R 5' – ACGCAGACTATCTTTCTAGGGTTAAAGACATGAC GATGCCCATGC -3'
P12		F 5' – CATCGTCATGTCTTTAACCCTAGAAAGATAGTC TGCG -3'
P13		R 5' – CGAGGCGCACCGTGGGCTTGTACTC -3'
P14		F 5' – CGTCACGCAGGAAAGGGACGAG -3'
P15		R 5' – CTCGTCCCTTTCCTGCGTGACG -3'
P16		F 5' – CTGGCACCCAATGGAAGCCATG -3'
P17		F 5' – ATGGCTTCCATTGGGTGCCAG -3'
P18	gRNA-B5 vector construction	F 5' – TTTCTTGGCTTTATATATCTTGTGGAAAGGA CGAAACACCGCCTTCTTGCTGGCACCCAA-3'
P19		R 5' – GACTAGCCTTATTTTAACTTGCTATTTCTAGCT CTAAAACTTGGGTGCCAGCAAGAAGGC-3'
OT1-A	gRNA-A1 off-target analysis	F 5' - CCTGAGCTGCTCTCCTTTCC -3'<br>R 5' – CCAGAGCATTGCCAAAGAGC -3'
OT2-A		F 5'- CAGCATGGAGAAGAGGAGCC -3'<br>R 5' – ACTGTCACCCTTGTCCCAGA -3'
OT3-A		F 5' – GATGCCATCATGGAGCCTCT -3'<br>R 5' – ACCCTAGTGACCAGCATGGA -3'
OT4-A		F 5'- TGCACTCAATGAGCAAGGCT -3'<br>R 5' – CCCAGCTGGACCAGGTAGTA -3'
OT5-A		F 5' - GCCATCTCCCTGTTCCTCAC -3'<br>R 5' – TCCCCATGCTTCTCACACAG -3'
OT1-B	gRNA-B5 off-target analysis	F 5'- ATGCAGCCCAGCTCAAGAAG -3'<br>R 5'- CGAAAGCTTCCGGCCTTGAG -3'
OT2-B		F 5'- GGAGTGCCAGGAGCACTAAC -3'<br>R 5'- CCAGAGGGCAACTGAGAGTG -3'
OT3-B		F 5' - GGACGCCCGACTCTTTAGTG -3'<br>R 5'- GCCTATTGACCCCTCACCTG -3'
OT4-B		F 5'- GTGGGAGAAGGAGAAAGACGAG -3'<br>R 5' - ACACTGCAAGTGACCTCCTG -3'

Table S1. continued

OT5-B		F 5' - GGACCACCAGGCTCTTCTTC -3'<br>R 5' - TGGCTCCCAACTCTGTAAGC -3'
OT6-B		F 5'- GGTGAGAGAGTTCGTCGTCC -3'<br>R 5' - GACCAGGGAGACAGTGAAGC -3'
OT7-B		F 5'- TCTCTGCACCCATGGAAACC -3'<br>R 5' - GCCCGCTTGTTCCCTAAGAT -3'
OT8-B		F 5' - CCCATGCCCTAATCCCATCC -3'<br>R 5' - GTGCAGCTGCTGGAATGAAG -3'
OT9-B		F 5' - TCTGAACTTTGCTCCGTGCT -3'<br>R 5' – AAGCCATTGCCATCCTCTCC -3'
OT10-B		F 5'- TGACCCTGTGCGAGAAGAAC-3'<br>R 5'- CAAGCCAGTCTGGGTAAGGG-3'
OT11-B		F 5'- TTCTCTCACTCCCTCCCCAG -3'<br>R 5'- TGTCTCAAGTGGGTGCTGTC-3'
P20	Q-PCR clones verification	F 5'- AATGGAAGCCATGCGCCGGA -3'
P21		F 5' – GCTGAGTGTGCAGGACGAGT -3'
P22		R 5'- ACGCTCGAACTTGGCAATGGCT -3'
P23	Reference primers for genomic Q-PCR	F 5'- CAGCTATTGGTGTGAGGGCA -3'
P24		R 5'- ATTTGTCGTTCCCAGCCTGT -3'
P25	Plasmid integration analysis	F 5' – TACCCGAGCCGATGACTTAC -3'
P26		R 5'- GCAACTAGAAGGCACAGTCG -3'
P27		F 5'-TACGATACAAGGCTGTTAGAGAG-3'
P28		R 5'- AAAAGCACCGACTCGGTGCC- 3'
P29		F 5' – CATGGACAAGAAGTACTCCATTG- 3'
P30		R 5' – GAACAAACGACCCAACACCCGTG – 3'
P31		F 5' – GAAGACAATAGCAGGCATGCTGG- 3'
P32		R 5' – AAGCCCTCCCGTATCGTAGT- 3'

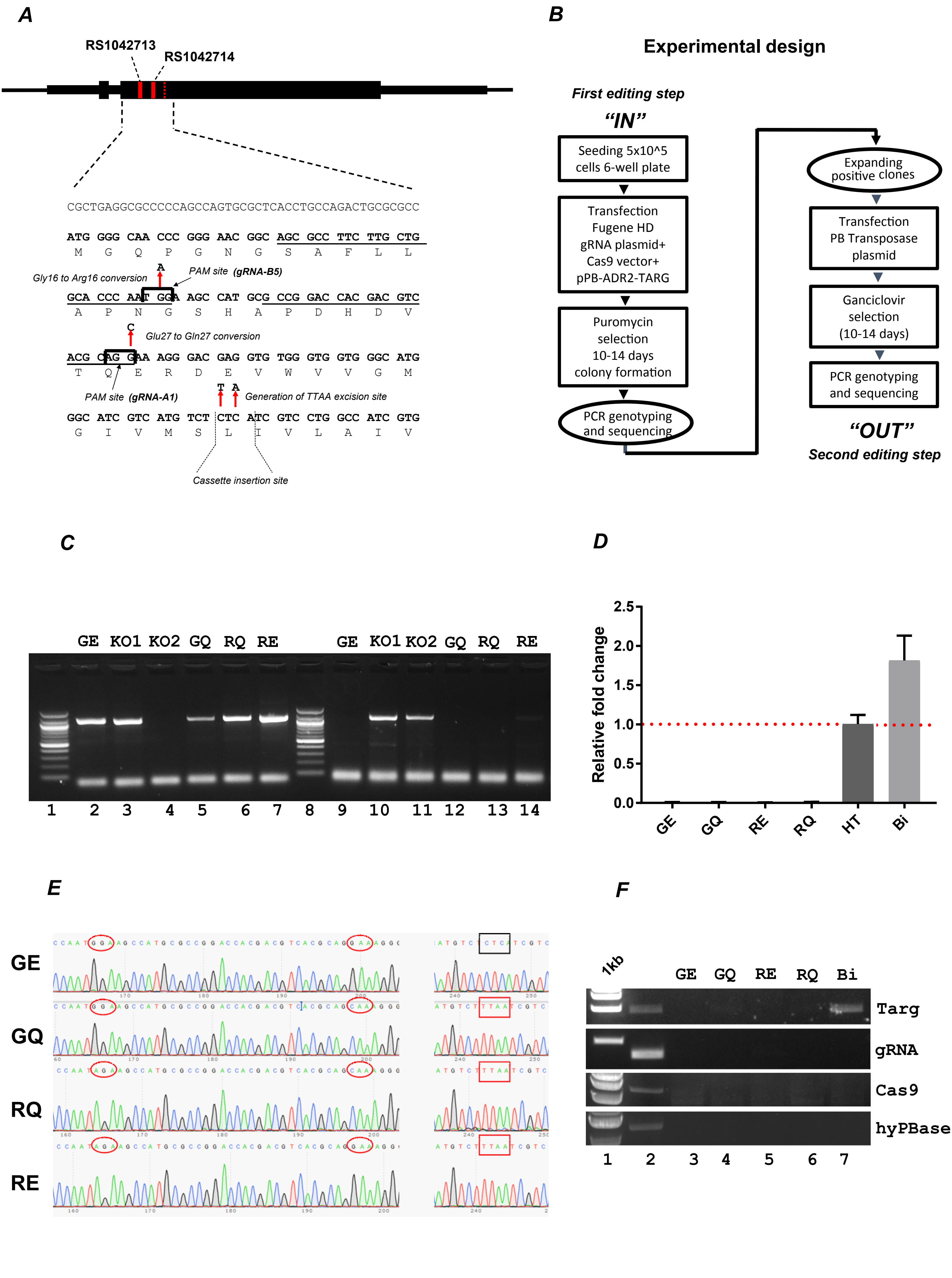
A
RS1042713
RS1042714
CGCTGAGGCGCCCCCAGCCAGTGCGCTCACCTGCCAGACTGCGCGCC
ATG GGG CAA CCC GGG AAC GGC AGC GCC TTC TTG CTG
M G Q P G N G S A F L L
Gly16 to Arg16 conversion
PAM site (gRNA-B5)
GCA CCC AAT GGA AGC CAT GCG CCG GAC CAC GAC GTC
A P N G S H A P D H D V
Glu27 to Gln27 conversion
ACG CAG GAA AGG GAC GAG GTG TGG GTG GTG GGC ATG
T Q E R D E V W V V G M
PAM site (gRNA-A1)
Generation of TTAA excision site
GGC ATC GTC ATG TCT CTC ATC GTC CTG GCC ATC GTG
G I V M S L I V L A I V
Cassette insertion site
B
Experimental design
First editing step
"IN"
Seeding 5x10^5 cells 6-well plate
Transfection Fugene HD gRNA plasmid+ Cas9 vector+ pPB-ADR2-TARG
Puromycin selection 10-14 days colony formation
PCR genotyping and sequencing
Expanding positive clones
Transfection PB Transposase plasmid
Ganciclovir selection (10-14 days)
PCR genotyping and sequencing
"OUT"
Second editing step
C
GE KO1 KO2 GQ RQ RE GE KO1 KO2 GQ RQ RE
1 2 3 4 5 6 7 8 9 10 11 12 13 14
D
Relative fold change
GE GQ RE RQ HT Bi
E
GE
GQ
RQ
RE
F
1kb
GE GQ RE RQ Bi
Targ
gRNA
Cas9
hyPBase
1 2 3 4 5 6 7

Figure S1

Figure S1 Experimental design and verification of targeted clones by PCR-genotyping and sequencing. A) Intronless structure of ADRB2 gene. Selected sequence shows location of polymorphic letters, gRNA recognition sequence (underlined) and PAM site (squared). Arrows indicate individual nucleotides changes for position 48 and 79 and for the introduction of TTAA site for cassette insertion; B) The outline of targeting experiment showing two main editing steps, the first step – CRISPR/Cas9 HDR mediated integration of PiggyBac selection cassette into ADRB2 locus (left), and second – PB transposase mediated cassette excision with recovery of functional ADRB2 gene structure (right). RQ variant was created with short-cut variant of this strategy[22], avoiding the first PCR-genotyping and sequencing step and directly transfecting Puro-resistant clones with PB transposase. C) On the Left panel, PCR analysis with P2 and P3 primers pair (see Fig. 1A) after cassette excision demonstrates recovery of ADRB2 locus in selected GQ, RQ and RE clones. HUES7 (GE) serves as positive control and biallelic clone before transposase excision step (KO2) as negative. KO1 is a heterozygous clone (before excision) with cassette inserted into one allele and indel in the other, the latter allele is amplified. The right panel demonstrates the absence of PiggyBac cassette. Primers pair P2 and P1 was used. As a positive control, clones KO1 and KO2 (see above) were used, showing the presence of cassette within ADRB2 locus. None of the final isogenic lines (GQ, RQ and RE) shows positive band; D) the results of Q-PCR (shown as means ±SD) with primer pairs P1 and P20 further confirms complete excision of PB cassette, it also shows the absence of remnants of targeting vector in the genome. PCR with controls heterozygous (HT) and biallelic targeted clone (Bi) demonstrates the presence of single or double copies of cassette accordingly; E) Shows sequencing results of the edited clones and demonstrates successful editing. Corrected codons shown by red circles. Also, excision of cassette can be seen by restoring TTAA excision site (highlighted by red square, compare to sequence from original background line, black squared); F) PCR of targeted lines after excision, and control biallelic targeted clone before excision step, with set of primers specific for: targeting vector (primers P25 and P26, TK gene), gRNA vector (P27 and P28 primer pair), Cas9 expressing vector (P29 and P30 primers, Cas9 gene) and hyPBtransposase expressing vector (P31 and P32 primer pair). The presence of TK gene can be seen in control sample (before excision). The absence of signal with selected regions demonstrates no random integrations of the latter in genome.

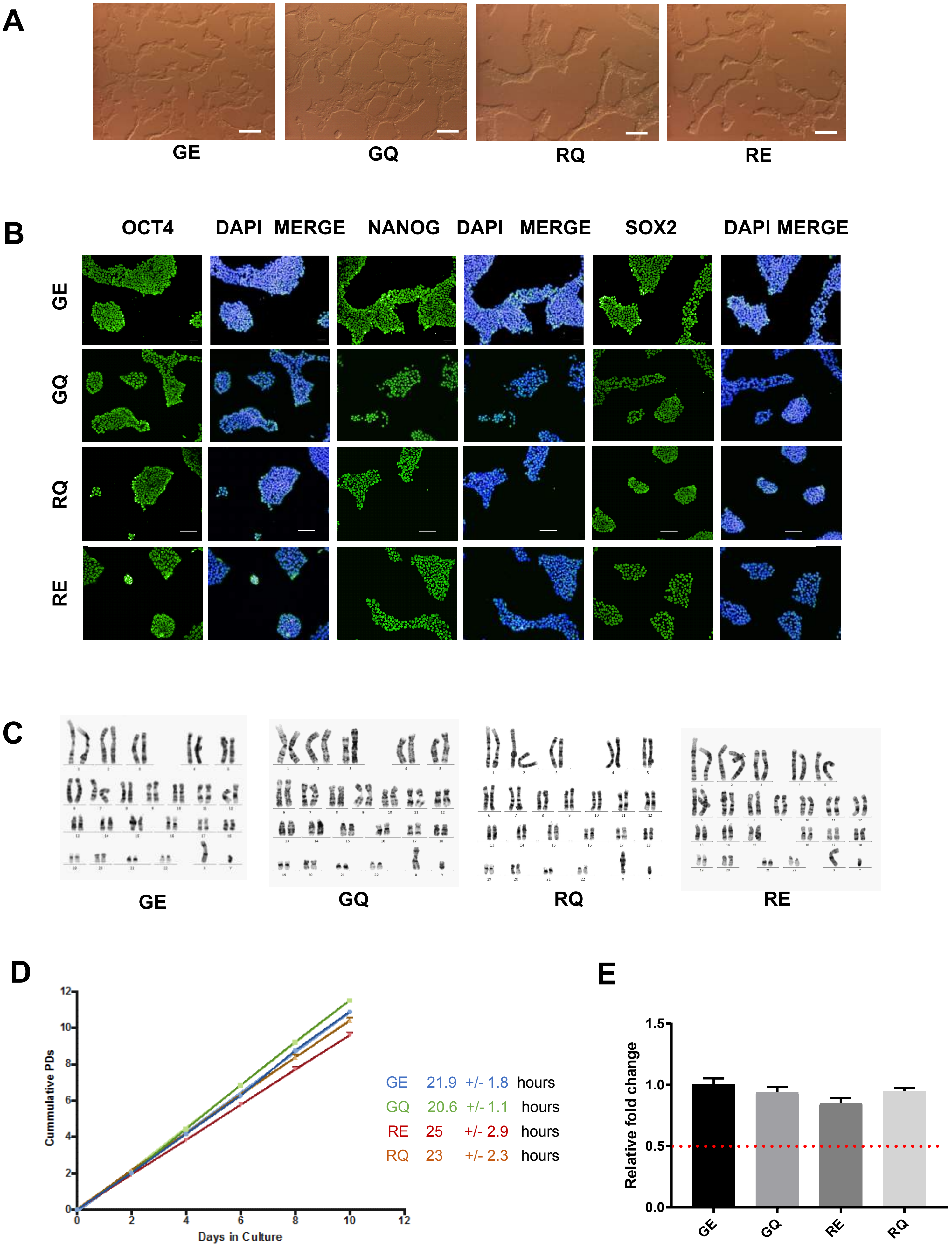
A
GE
GQ
RQ
RE
B
OCT4
DAPI MERGE
NANOG
DAPI
MERGE
SOX2
DAPI MERGE
GE
GQ
RQ
RE
C
GE
GQ
RQ
RE
D
Cummulative PDs
Days in Culture
GE 21.9 +/- 1.8 hours
GQ 20.6 +/- 1.1 hours
RE 25 +/- 2.9 hours
RQ 23 +/- 2.3 hours
E
Relative fold change
GE
GQ
RE
RQ

Figure S2

Figure S2 Assessment of undifferentiated edited hPSC for pluripotency, karyotype abnormalities and growth characteristics.
A) Immunostaining for the pluripotency markers OCT4, NANOG and SOX2 (green columns), DAPI was used to localise nuclei (blue). All edited lines (GQ, RE and RQ) as well as background HUES7 line (GE) retain their pluripotency characteristics; G-banding karyotyping of 30 metaphase spreads per line, with a representative karyogram shown for each line shown in panel (B) and demonstrates no abnormalities; panel (C) shows similar growth pattern for each line; panel (E) shows comparable Q-PCR signal (shown as means±SD) with ADRB2 locus specific primers (P21 and P22) between all isogenic lines. No difference can be seen between edited and parental (GE) line. This result support the absence multiplication (or large deletions of a single allele) of the targeted locus in the genome.

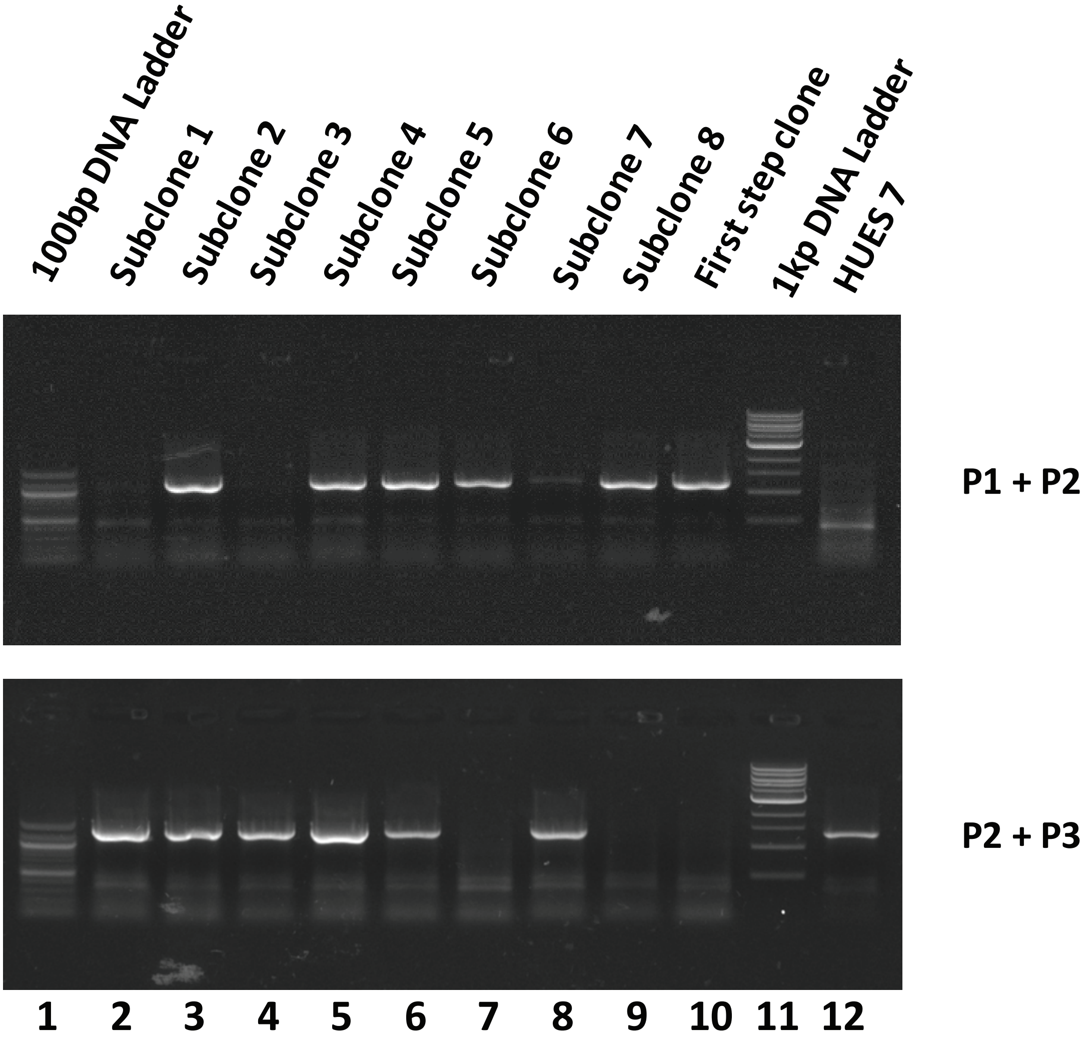

Figure S3 Example of clonal screening (GQ editing clones) after excision step. Showing presence of three type of colonies: completely excised (lines NN 2, 4 and 8); one allele excised with second carrying insert ("heterozygous" for excision, see lines NN 3, 5, 6); not excised, similar to parental clone with both alleles carrying insert. Note, that clones in lines 2 to 5 derived from parental clone shown on line 10. The presence of "heterozygous" for excision clones confirms the absence of large deletion in this case.

Figure S3

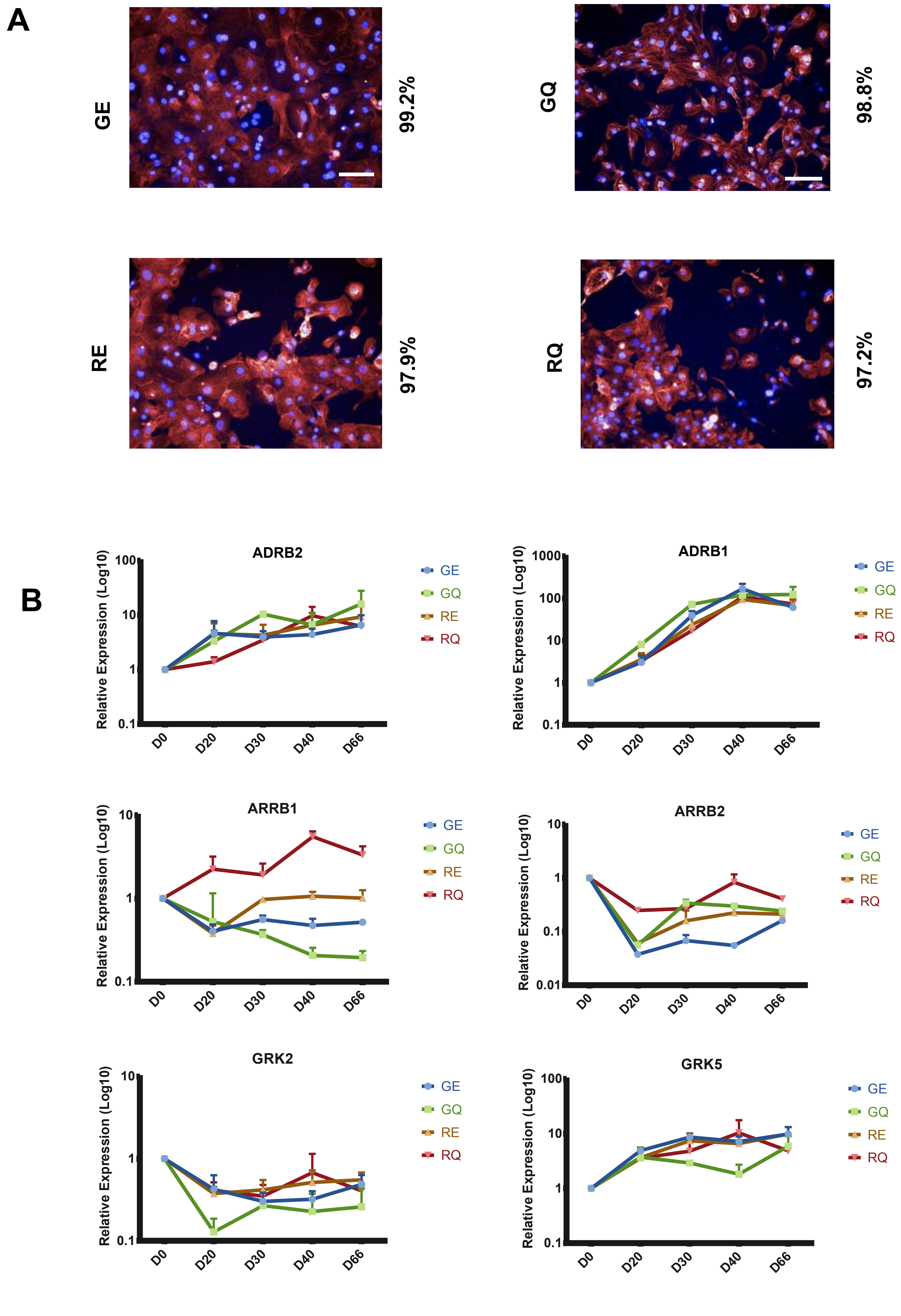
A
GE
99.2%
GQ
98.8%
RE
97.9%
RQ
97.2%
B
ADRB2
ADRB1
ARRB1
ARRB2
GRK2
GRK5
Relative Expression (Log10)
D0
D20
D30
D40
D66
GE
GQ
RE
RQ

Figure S4

Figure S4 Edited polymorphism-specific lines can be differentiated to cardiomyocytes and express components of adrenergic system. A) Cells were differentiated by direct monolayer differentiation protocol (22), Immunostaining for α-actinin (red) demonstrates more than 90% purity of cardiomyocytes (estimated as relation to total nuclei count, DAPI, blue); B) time dependent expression of the selected mRNAs related to β-adrenergic signalling (ADRB2, ADRB1, GRK5 & GRK2) gene was evaluated by quantitative real-time PCR in four isogenic lines from d0 to d66 of differentiation. For each line, all of the samples are normalised to 18SRNA and data from two independent batches of differentiation, each measured x3 times are shown as a mean±SEM relative to d0 (undifferentiated).

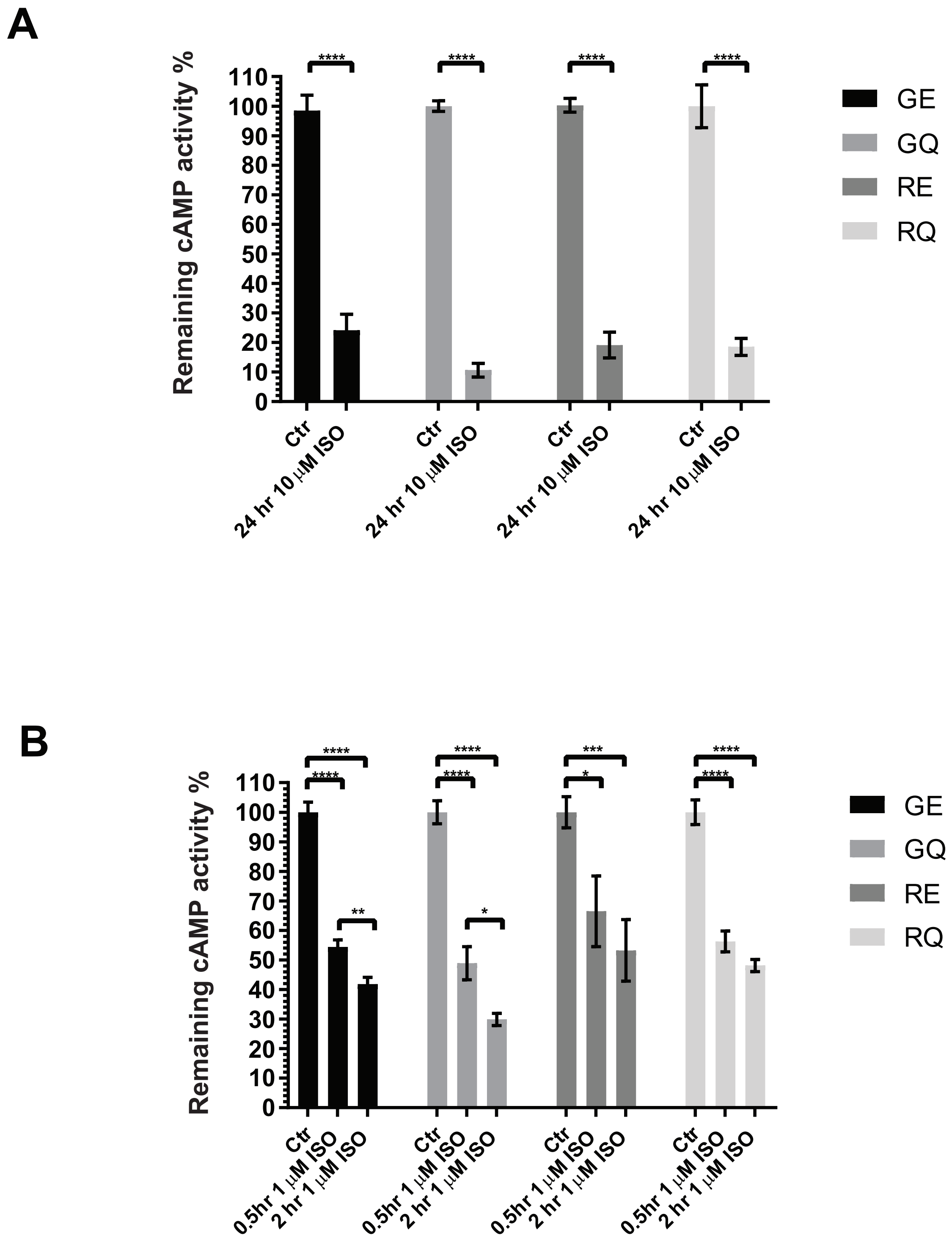

Figure S5 Desensitisation after long- and short-term pre-treatment with isoprenaline compare to untreated control. Significant desensitisation can be seen in edited polymorphism specific lines after 24 hr pre-treatment with 10 μM isoprenaline (panel A) or 0.5/2hr pre-treatment with 1 μM isoprenaline compare to untreated control cells (panel B). Data are presented as means ± SEM ($n \geq 7$, two-tailed unpaired t-test, *p= 0.0206, **p=≤0.0039, ***p=0.001 and ****p≤0.0001).

Figure S5

A

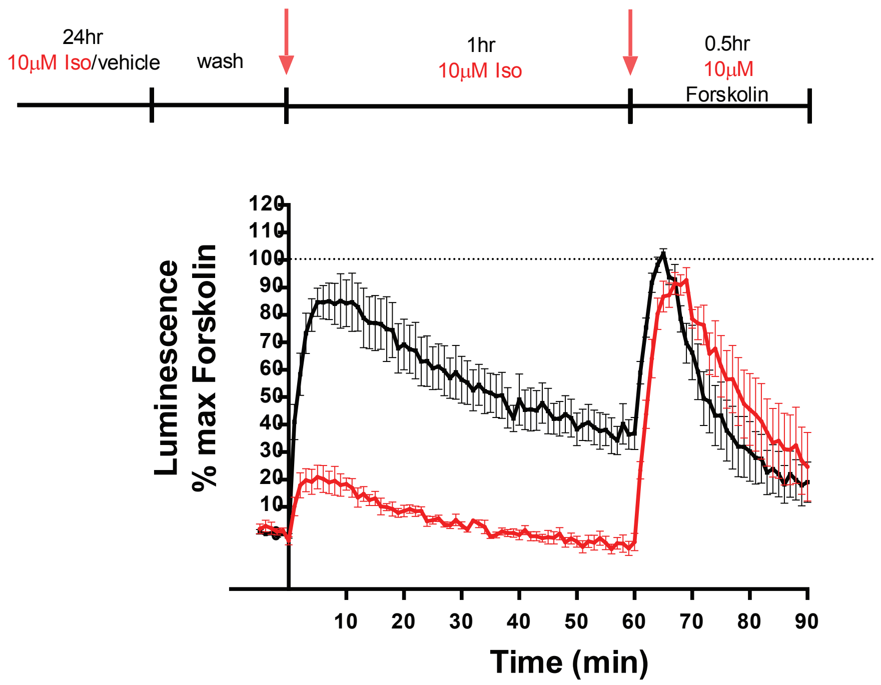

B

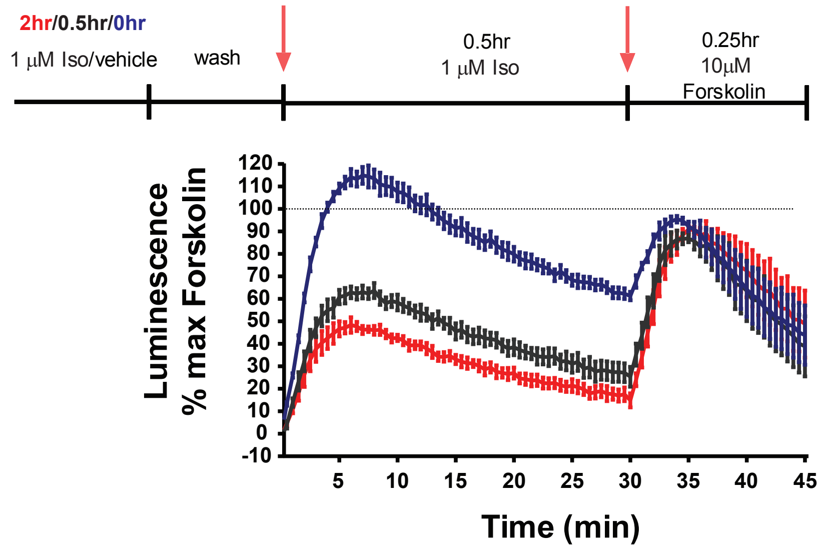

Figure S6 Representative traces to show experiments design and outline of selected traces for downregulation. (panel A) and desensitisation (panel B) experiments. Arrows indicates addition of Isoprenaline or forskolin.

Figure S6

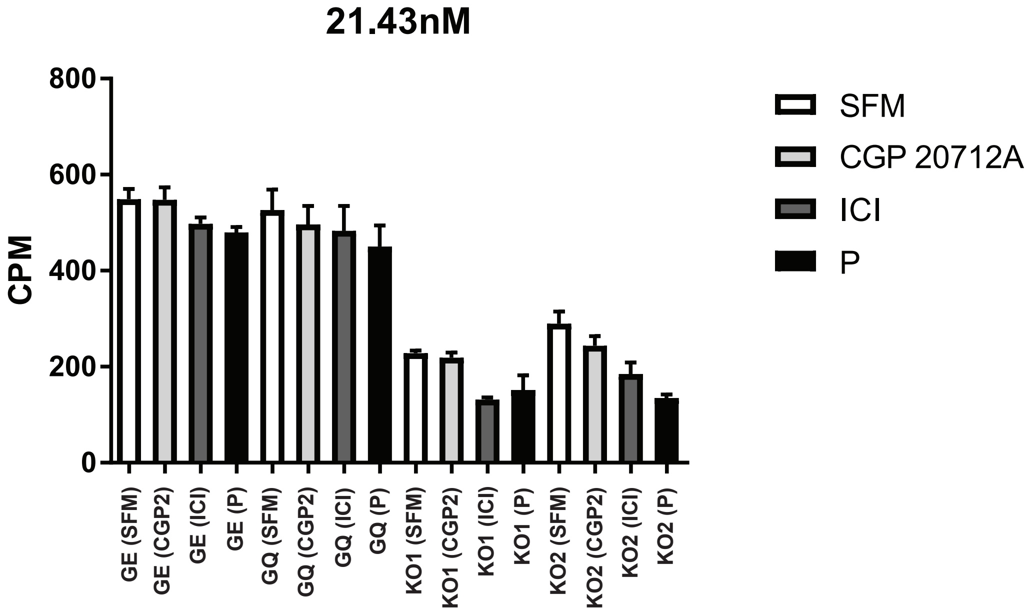

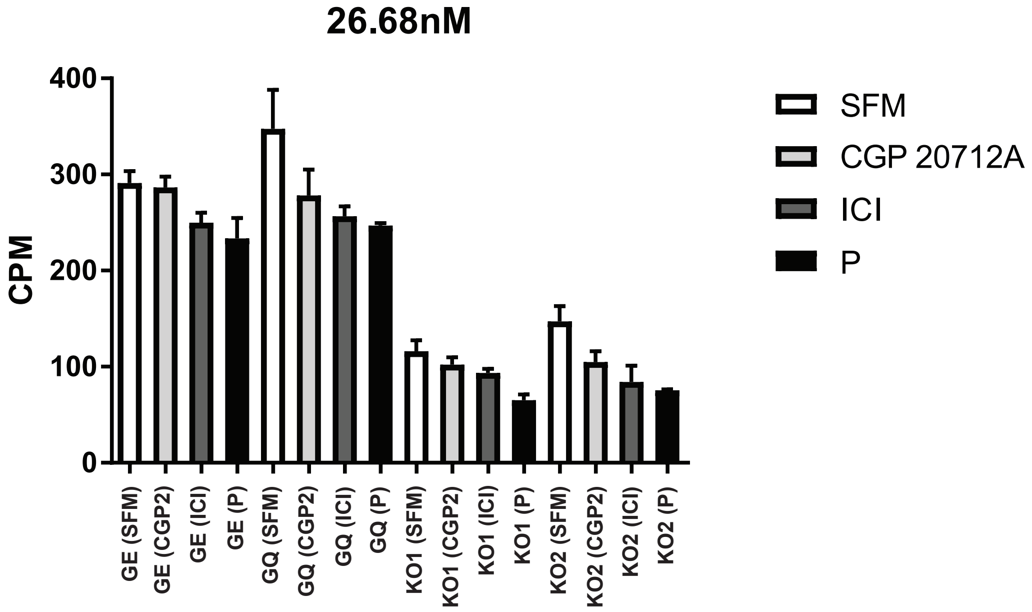

Figure S7 Radioligand assay raw data from 2 proof-of-principle experiments. 4 cells lines were examined - two with differing SNPs (GQ and GE) and two which were beta2-adrenergic knockout lines (KO1 and KO2). Binding of 3H-CGP 12177 in untreated cells (SFM: serum free media alone) was low; 200-600 CPM (counts per minute). Pre-incubation with a high concentration of propranolol (P, 10 µM) to determine non-specific binding illustrated that our measurement window for determining specific binding was very small – it would be very difficult to assess specific binding above non-specific. This is emphasised by the small displacement of binding observed in the knockout lines when challenged with the beta2-selective antagonist ICI 118551.

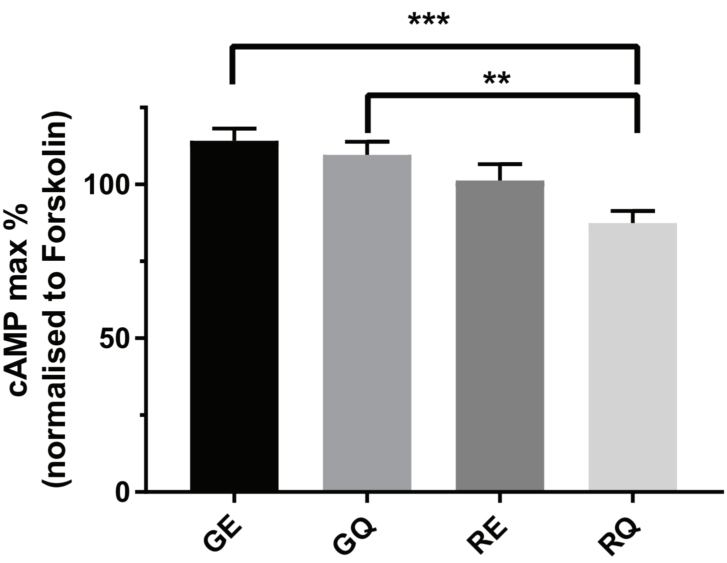

Figure S8 Comparison of max β2AR-mediated cAMP response induced by 1 µM isoprenaline in β2AR variant-specific cardiomyocytes in presence of 0.1 µM CGP20172A. Data presented as percentage of max forskolin response (shown as means ± SEM; n ≥ 7, **p=0.0031, **p=0.0035, one-way ANOVA with Tukey's multiple comparisons test)

Figure S8

pPB-B2-TARG-GE targeting vector sequence

ctaaattgtaagcgttaatattttgttaaaattcgcgttaaatttttgttaaatcagctcattttttaaccaataggccgaaatcggcaaaatcccttataaatcaaaagaatagaccgagatagg gttgagtgttgttccagtttggaaca
agagtccactattaaagaacgtggactccaacgtcaaagggcgaaaaaccgtctatcagggcgatggcccactacgtgaaccatcaccctaatcaagttttttgg ggtcgaggtgccgtaaagcactaaatcggaaccctaaaggga
gcccccgatttagagcttgacggggaaagccggcgaacgtggcgagaaaggaagggaagaaagcgaaaggagcgggcgctagggcgctggcaagtgtagcggtcacgctgcgcgtaaccaccaca cccgccgcgcttaatgcgcc
gctacagggcgcgtcccattcgccattcaggctgcgcaactgttgggaagggcgatcggtgcgggcctc ttcgctattacgccagctggcgaaagggggatgtgctgcaaggcgattaagttgggtaacgccagggttttcccagtcac
gacgttgtaaaacgacggccagtgagcgcgcgtaatacgactcactatagggcgaattgggtaccgggccccccctcgaggtcgacggtatcgataagcttgatTTCGGAGTACCCAGATGGAGACATCCGTGTCTGT
GTCGCTCTGGATGCCTCCAAGCCAGCGTGTGTTTACTTTCTGTGTGTGTCACCATGTCTTTGTGCTTCTGGGTGCTTCTGTGTTTGTTTCTGGCCGCGTTTCTGTGTTGGACAGGGG
TGACTTTGTGCCGGATGGCTTCTGTGTGAGAGCGCGCGCGAGTGTGCATGTCGGTGAGCTGGGAGGGTGTGTCTCAGTGTCTATGGCTGTGGTTCGGTATAAGTCTGAGCATGT
CTGCCAGGGTGTATTTGTGCCTGTATGTGCGTGCCTCGGTGGGCACTCTCGTTTCCTTCCGAATGTGGGGCAGTGCCGGTGTGCTGCCCTCTGCCTTGAGACCTCAAGCCGCGCA
GGCGCCCAGGGCAGGCAGGTAGCGGCCACAGAAGAGCCAAAAGCTCCCGGGTTGGCTGGTAAGGACACCACCTCCAGCTTTAGCCCTCTGGGGCCAGCCAGGGTAGCCGGGA
AGCAGTGGTGGCCCGCCCTCCAGGGAGCAGTTGGGCCCCGCCCGGGCCAGCCCCAGGAGAAGGAGGGCGAGGGGAGGGGAGGGAAAGGGGAGGAGTGCCTCGCCCCTTCG
CGGCTGCCGGCGTGCCATTGGCCGAAAGTTCCCGTACGTCACGGCGAGGGCAGTTCCCCTAAAGTCCTGTGCACATAACGGGCAGAACGCACTGCGAAGCGGCTTCTTCAGAGC
ACGGGCTGGAACTGGCAGGCACCGCGAGCCCCTAGCACCCGACAAGCTGAGTGTGCAGGACGAGTCCCCACCACACCCACACCACAGCCGCTGAATGAGGCTTCCAGGCGTCC
GCTCGCGGCCCGCAGAGCCCCGCCGTGGGTCCGCCCGCTGAGGCGCCCCCAGCCAGTGCGCTCACCTGCCAGACTGCGCGCCATGGGGCAACCCGGGAACGGCAGCGCCTTCT
TGCTGGCACCCAATGGAAGCCATGCGCCGGACCACGACGTCACGCAGGAAAGGGACGAGGTGTGGGTGGTGGGCATGGGCATCGTCATGTCT*TTAACCCTAGAAAGATAGTCT*
GCGTAAAATTGACGCATGCATTCTTGAAATATTGCTCTCTCTTTCTAAATAGCGCGAATCCGTCGCTGTGCATTTAGGACATCTCAGTCGCCGCTTGGAGCTCCCGTGAGGCGTGCT
TGTCAATGCGGTAAGTGTCACTGATTTTGAACTATAACGACCGCGTGAGTCAAAATGACGCATGATTATCTTTTACGTGACTTTTAAGATTTAACTCATACGATAATTATATTGTTAT
*TTCATGTTCTACTTACGTGATAACTTATTATATATATATATTTTCTTGTTATAGATATCAACTAGAATGCTAGC*ACAAGTTTGTACAAAAAAGCAGGCTGGCGCCGGAACCAATTCAGT
CGACTGGATCCGGTACCGGGCCCCCCCTCGAGGTCGAGACGGTATCGATAAGCTTGATATCGAATAATTCTACCGGGTAGGGGAGGCGCTTTTCCCAAGGCAGTCTGGAGCATG
CGCTTTAGCAGCCCCGCTGGGCACTTGGCGCTACACAAGTGGCCTCTGGCCTCGCACACATTCCACATCCACCGGTAGGCGCCAACCGGCTCCGTTCTTTGGTGGCCCCTTCGCGC
CACCTTCTACTCCTCCCCTAGTCAGGAAGTTCCCCCCCGCCCCGCAGCTCGCGTCGTGCAGGACGTGACAAATGGAAGTAGCACGTCTCACTAGTCTCGTGCAGATGGACAGCAC
CGCTGAGCAATGGAAGCGGGTAGGCCTTTGGGGCAGCGGCCAATAGCAGCTTTGCTCCTTCGCTTTCTGGGCTCAGAGGCTGGGAAGGGGTGGGTCCGGGGGCGGGCTCAGG
GGCGGGCTCAGGGGCGGGGCGGGCGCCCGAAGGTCCTCCGGAGGCCCGGCATTCTGCACGCTTCAAAAGCGCACGTCTGCCGCGCTGTTCTCCTCTTCCTCATCTCCGGGCCTT
TCGACCTGCAGCCTGTTGACAATTAATCATCGGCATAGTATATCGGCATAGTATAATACGACAAGGTGAGGAACTAAACC**ATGGGGACCGAGTACAAGCCCACGGTGCGCCTCG**
CCACCCGCGACGACGTCCCCCGGGCCGTACGCACCCTCGCCGCCGCGTTCGCCGACTACCCCGCCACGCGCCACACCGTCGACCCGGACCGCCACATCGAGCGGGTCACCGAG
CTGCAAGAACTCTTCCTCACGCGCGTCGGGCTCGACATCGGCAAGGTGTGGGTCGCGGACGACGGCGCCGCGGTGGCGGTCTGGACCACGCCGGAGAGCGTCGAAGCGGGG
GCGGTGTTCGCCGAGATCGGCCCGCGCATGGCCGAGTTGAGCGGTTCCCGGCTGGCCGCGCAGCAACAGATGGAAGGCCTCCTGGCGCCGCACCGGCCCAAGGAGCCCGCG
TGGTTCCTGGCCACCGTCGGCGTCTCGCCCGACCACCAGGGCAAGGGTCTGGGCAGCGCCGTCGTGCTCCCCGGAGTGGAGGCGGCCGAGCGCGCCGGGGTGCCCGCCTTCC
TGGAGACCTCCGCGCCCCGCAACCTCCCCTTCTACGAGCGGCTCGGCTTCACCGTCACCGCCGACGTCGAGGTGCCCGAAGGACCGCGCACCTGGTGCATGACCCGCAAGCCC
GGTGCCGGATCCATGCCCACGCTACTGCGGGTTTATATAGACGGTCCTCACGGGATGGGGAAAACCACCACCACGCAACTGCTGGTGGCCCTGGGTTCGCGCGACGATATCG
TCTACGTACCCGAGCCGATGACTTACTGGCAGGTGCTGGGGGCTTCCGAGACAATCGCGAACATCTACACCACACAACACCGCCTCGACCAGGGTGAGATATCGGCCGGGGA
CGCGGCGGTGGTAATGACAAGCGCCCAGATAACAATGGGCATGCCTTATGCCGTGACCGACGCCGTTCTGGCTCCTCATATCGGGGGGGAGGCTGGGAGCTCACATGCCCCG
CCCCCGGCCCTCACCCTCATCTTCGACCGCCATCCCATCGCCGCCCTCCTGTGCTACCCGGCCGCGCGATACCTTATGGGCAGCATGACCCCCCAGGCCGTGCTGGCGTTCGTGG
CCCTCATCCCGCCGACCTTGCCCGGCACAAACATCGTGTTGGGGGCCCTTCCGGAGGACAGACACATCGACCGCCTGGCCAAACGCCAGCGCCCCGGCGAGCGGCTTGACCTG
GCTATGCTGGCCGCGATTCGCCGCGTTTACGGGCTGCTTGCCAATACGGTGCGGTATCTGCAGGGCGGCGGGTCGTGGCGGGAGGATTGGGGACAGCTTTCGGGGACGGCC
GTGCCGCCCCAGGGTGCCGAGCCCCAGAGCAACGCGGGCCCACGACCCCATATCGGGGACACGTTATTTACCCTGTTTCGGGCCCCCGAGTTGCTGGCCCCCAACGGCGACCT
GTACAACGTGTTTGCCTGGGCCTTGGACGTCTTGGCCAAACGCCTCCGTCCCATGCACGTCTTTATCCTGGATTACGACCAATCGCCCGCCGGCTGCCGGGACGCCCTGCTGCA
ACTTACCTCCGGGATGGTCCAGACCCACGTCACCACCCCCGGCTCCATACCGACGATCTGCGACCTGGCGCGCACGTTTGCCCGGGAGATGGGGGAGGCTAACTGAGCTCTAG
AGCTCGCTGATCAGCCTCGACTGTGCCTTCTAGTTGCCAGCCATCTGTTGTTTGCCCCTCCCCCGTGCCTTCCTTGACCCTGGAAGGTGCCACTCCCACTGTCCTTTCCTAATAAAAT
GAGGAAATTGCATCGCATTGTCTGAGTAGGTGTCATTCTATTCTGGGGGGTGGGGTGGGGCAGGACAGCAAGGGGGAGGATTGGGAAGACAATAGCAGGCATGCTGGGGAT
GCGGTGGGCTCTATGGCTTCTGAGGCGGAAAGAACCAGCTGGGGCTCGAGATCCACTAGTTCTAGCCTCGAGGCTAGAGCGGCCGCACTCGAGATATCTAGACCCAGCTTTCTT
GTACAAAGTGGT*ACTAGTTAAAAGTTTTGTTACTTTATAGAAGAAATTTTGAGTTTTTGTTTTTTTTTAATAAATAAATAAACATAAATAAATTGTTTGTTGAATTTATTATTAGTAT*
GTAAGTGTAAATATAATAAAACTTAATATCTATTCAAATTAATAAATAAACCTCGATATACAGACCGATAAAACACATGCGTCAATTTTACGCATGATTATCTTTAACGTACGTCAC
*AATATGATTATCTTTCTAGGGTTA*ATCGTCCTGGCCATCGTGTTTGGCAATGTGCTGGTCATCACAGCCATTGCCAAGTTCGAGCGTCTGCAGACGGTCACCAACTACTTCATCACT
TCACTGGCCTGTGCTGATCTGGTCATGGGCCTGGCAGTGGTGCCCTTTGGGGCCGCCCATATTCTTATGAAAATGTGGACTTTTGGCAACTTCTGGTGCGAGTTTTGGACTTCCAT
TGATGTGCTGTGCGTCACGGCCAGCATTGAGACCCTGTGCGTGATCGCAGTGGATCGCTACTTTGCCATTACTTCACCTTTCAAGTACCAGAGCCTGCTGACCAAGAATAAGGCC
CGGGTGATCATTCTGATGGTGTGGATTGTGTCAGGCCTTACCTCCTTCTTGCCCATTCAGATGCACTGGTACCGGGCCACCCACCAGGAAGCCATCAACTGCTATGCCAATGAGAC
CTGCTGTGACTTCTTCACGAACCAAGCCTATGCCATTGCCTCTTCCATCGTGTCCTTCTACGTTCCCCTGGTGATCATGGTCTTCGTCTACTCCAGGGTCTTTCAGGAGGCCAAAAG
GCAGCTCCAGAAGATTGACAAATCTGAGGGCCGCTTCCATGTCCAGAACCTTAGCCAGGTGGAGCAGGATGGGCGGACGGGGCATGGACTCCGCAGATCTTCCAAGTTCTGCTT
GAAGGAGCACAAAGCCCTCAAGACGTTAGGCATCATCATGGGCACTTTCACCCTCTGCTGGCTGCCCTTCTTCATCGTTAACATTGTGCATGTGATCCAGGATAACCTCATCCGTA
AGGAAGTTTACATCCTCCTAAATTGGATAGGCTATGTCAATTCTGGTTTCAATCCCCTTATCTACTGCCGGAGCCCAGATTTCAGGATTGCCTTCCAGGAGCTTCTGTGCCTGCGCA
GGTCTTCTTTGAAGGCCTATGGGAATGGCTACTCCAGCAACGGCAACACAGGGGAGCAGAGTGGATATCACGTGGAACAGGAGAAAGAAAATAAACTGCTGTGTGAAGACCTC
CCAGGCACGGAAGACTatcgaattcctgcagcccgggggatccactagttctagagcggccgccaccgcggtggagctccagcttttgttccctttagtgagggttaattgcgcgcttggcgtaatcatggtcatagctgt ttcctg
tgtgaaattgttatccgctcacaattccacacaacatacgagccggaagcataaagtgtaaagcctggggtgcctaatgagtgagctaactcacattaattgcgttgcgctcactgcccgctttccagtcgggaaac ctgtcgtgccagct
gcattaatgaatcggccaacgcgcggggagaggcggtttgcgtattgggcgctcttccgcttcctcgctcactgactcgctgcgctcggtcgttcggctgcggcgagcggtatcagc tcactcaaaggcggtaatacggttatccacaga
atcaggggataacgcaggaaagaacatgtgagcaaaaggccagcaaaaggccaggaaccgtaaaaaggccgcgttgctggcgtttttccataggctccgcccccctgacgagcatcacaaaaatc gacgctcaagtcagaggtggc
gaaacccgacaggactataaagataccaggcgtttccccctggaagctccctcgtgcgctctcctgttccgaccctg ccgcttaccggatacctgtccgcctttctcccttcgggaagcgtggcgctttctcatagctcacgctgtaggtat
ctcagttcggtgtaggtcgttcgctccaagctgggctgtgtgcacgaaccccccgttcagcccgaccgctgcgccttatccggtaactatcgtcttgagtccaacccggtaagacacgacttatc gccactggcagcagccactggtaaca
ggattagcagagcgaggtatgtaggcggtgctacagagttcttgaagtggtggcctaactacggctacactagaaggacagtatttggtatctgcgctctgctgaagccagttaccttcggaaaaagagttggtagctcttgatccggca
aacaaaccaccgctggtagcggtggtttttttgtttgcaagcagcagattacgcgcagaaaaaaaggatctcaagaagatcctttgatcttttctacggggtctgacgctcagtggaacgaaaac tcacgttaagggattttggtcatga
gattatcaaaaaggatcttcacctagatccttttaaattaaaaatgaagttttaaatcaatctaaagtatatatgagtaaacttggtctgacagttaccaatgcttaatcagtgaggcacctatc tcagcgatctgtctatttcgttcatcca
tagttgcctgactccccgtcgtgtagataactacgatacgggagggcttaccatctggccccagtgctgcaatgataccgcgagacccacgctcaccggctccagatttatcagcaataaaccagccagccggaagggccgagcgcag
aagtggtcctgcaactttatccgcctccatccagtctattaattgttgccgggaagctagagtaagtagttcgccagttaatagtttgcgcaacgttgttgccattgctacaggcatcgtggtgt cacgctcgtcgtttggtatggcttcattc
agctccggttcccaacgatcaaggcgagttacatgatcccccatgttgtgcaaaaaagcggttagctccttcggtcctccgatcgttgtcagaagtaagttggccgcagtgttatcactcatggttatggcagcactgcataattctcttac
tgtcatgccatccgtaagatgcttttctgtgactggtgagtactcaaccaagtcattctgagaatagtgtatgcggcgaccgagttgctcttgcccggcgtcaatacgggataataccgcgccac atagcagaactttaaaagtgctcatc
attggaaaacgttcttcggggcgaaaactctcaaggatcttaccgctgttgagatccagttcgatgtaacccactcgtgcacccaactgatcttcagcatcttttactttcaccagcgtttctgg gtgagcaaaaacaggaaggcaaaatg
ccgcaaaaaagggaataagggcgacacggaaatgttgaatactcatactcttcctttttcaatattattgaagcatttatcagggttattgtctcatgagc ggatacatatttgaatgtatttagaaaaataaacaaataggggttccgcg
cacatttccccgaaaagtgccac

Figure S9

Figure S9 Complete sequence of pPB-B2-TARG-GE vector shown. Small letters correspond to background plasmid sequence; underlined are left (top) and right (bottom) homology arms for ADRB2 locus; PiggyBac terminal repeats are shown in italic and puroΔTK coding sequence is highlighted in bold. The positions of polymorphic letters in the left arm of homology is indicated by red. The GQ, RQ and RE targeting vectors were made on the basis of this sequence and identical to it, apart of corresponding changes in indicated polymorphic positions.

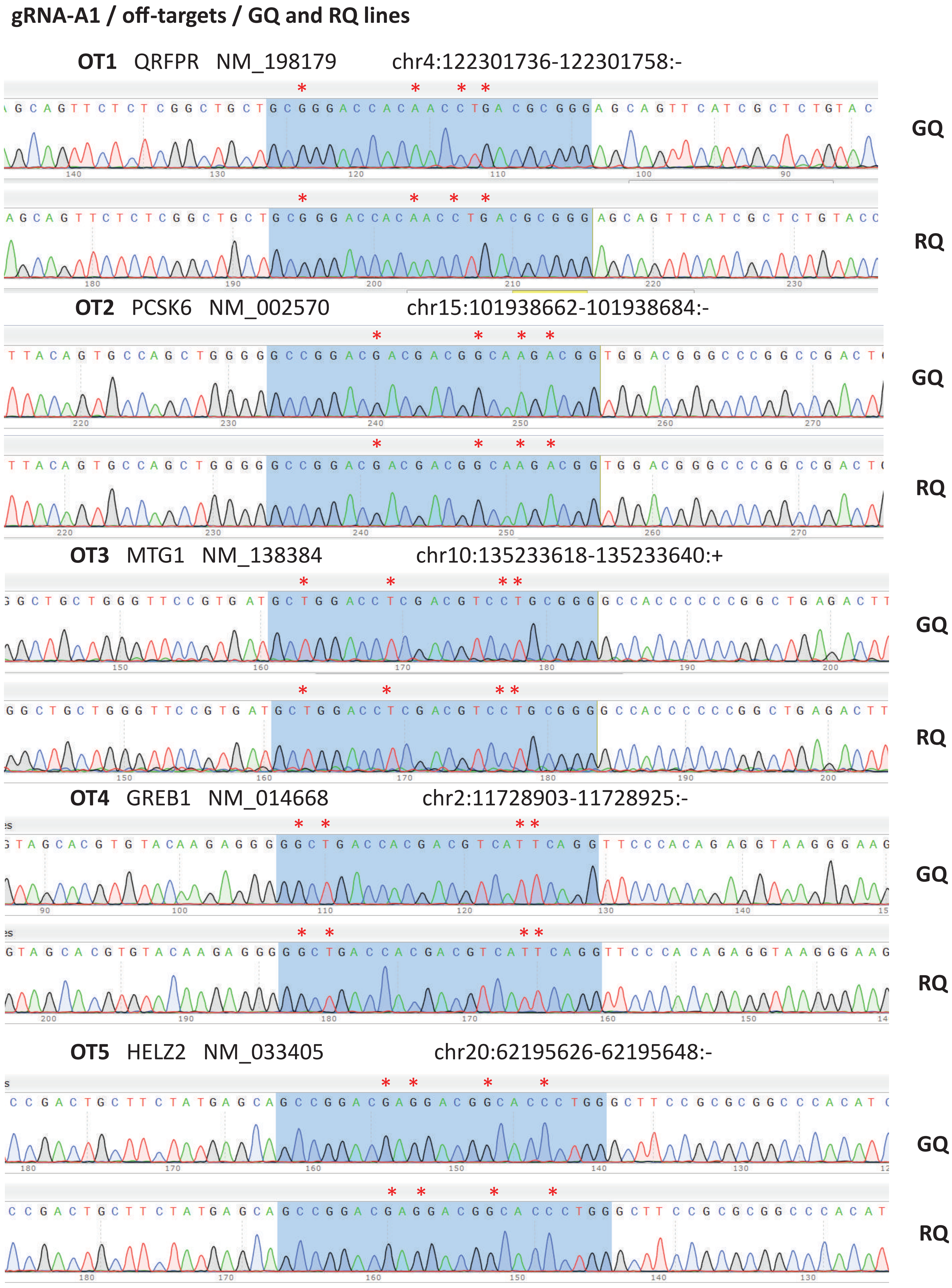

Figure S10 Analysis for off-target modifications in lines (GQ and RQ) created with gRNA-A1 vector. No modifications were observed in selected off-target sites. Gene names and positions of predicted recognition sites are indicated.

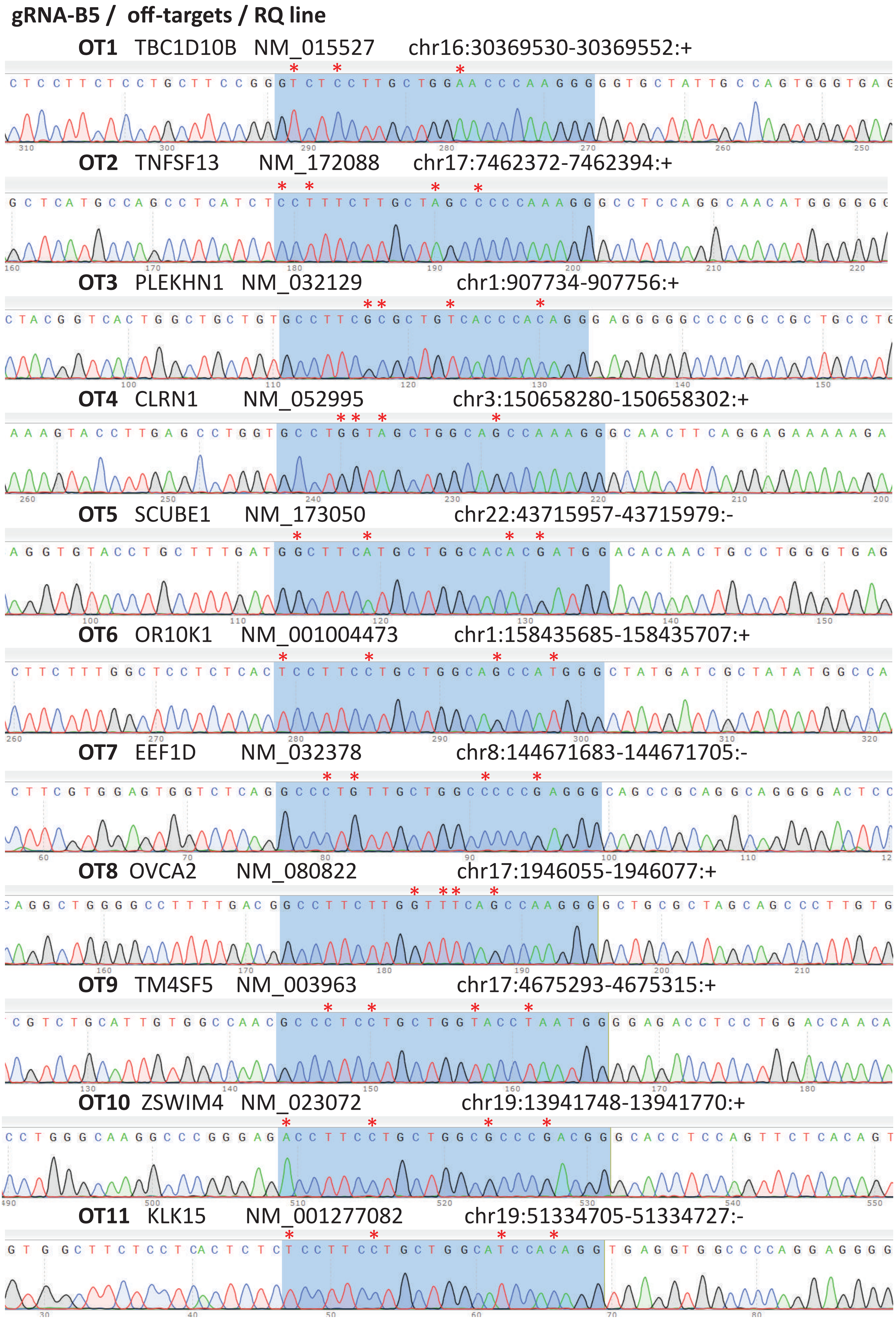

Figure S11 Analysis for off-target modifications in RE line created with gRNA-B5 vector. No modifications were observed in selected off-target sites. Gene names and positions of predicted recognition sites are indicated.
